# Revealing and Mitigating Crossover-Driven Side Reactions in Ferrocyanide-Based Redox Flow Batteries

**DOI:** 10.1021/acselectrochem.5c00178

**Published:** 2025-08-14

**Authors:** Emma J. Latchem, Thomas Kress, Muireann Anna de h-Óra, Anqi Wang, Qilei Song, Alexander C. Forse

**Affiliations:** † Yusuf Hamied Department of Chemistry, 2152 University of Cambridge, Lensfield Rd, Cambridge CB2 1EW, United Kingdom; ‡ Department of Materials Science and Metallurgy, 2152 University of Cambridge, Charles Babbage Rd, Cambridge CB3 0FS, United Kingdom; § Barrer Centre, Department of Chemical Engineering, 4615 Imperial College London, London SW7 2AZ, United Kingdom; ∥ Physical Science and Engineering Division, King Abdullah University of Science and Technology, Thuwal 23955-6900, Saudi Arabia

**Keywords:** Redox flow batteries, aqueous batteries, electrolyte crossover, electrolyte degradation, on-line NMR spectroscopy, ferricyanide, ferrocyanide, quinone

## Abstract

There is an urgent need for new energy storage solutions that will support the decarbonization of the electricity grid. Aqueous organic redox flow batteries are low-cost, long-duration energy storage devices that are in the process of being commercialized for this application; however, their operational lifetime is limited by electrolyte decomposition and crossover. These degradation processes are generally studied separately, so the relationship between the two is poorly understood. Previously, it had been assumed that the main contribution to battery capacity fade was electrochemical degradation of the electrolytes. Using the on-line ^1^H NMR crossover characterization method we developed previously, we reveal the first experimental evidence for crossover-driven side reactions in redox flow batteries. If the impact of these side reactions is not considered, it will lead to an underestimation of crossover and its impacts on battery lifetime. We further introduce simple ‘simulated-crossover’ experiments to identify anolyte-catholyte combinations where these processes are occurring. Using these simulated-crossover experiments, we find that crossover-driven side reactions can be mitigated by avoiding the use of anolytes with hydroxyl functional groups when using ferrocyanide electrolytes. These insights should be used to assist the design of new anolytes and catholytes, which will facilitate the development of longer-lasting redox flow batteries.

## Graphical abstract

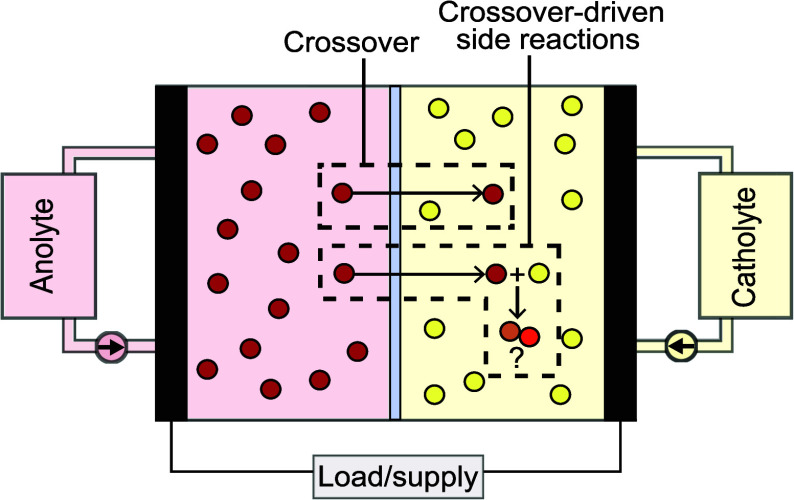

## Introduction

An aqueous organic redox flow battery (AORFB) is a type of redox flow battery that utilizes organic redox-active material, dissolved in an aqueous electrolyte.^1–6^ The battery is comprised of two liquid electrolytes, the anolyte and the catholyte, also known as the negolyte and posolyte, respectively.^7–9^ These electrolytes are stored externally in reservoirs and are pumped into an electrochemical cell to be charged, and later discharged.^5,8,9^ The anolyte and catholyte are separated by an ion-selective membrane, which is designed to conduct charge-balancing ions, whilst preventing the mixing of the redox-active material.^7,10–13^ As the energy is stored in solution, rather than in the electrode, the capacity of these devices is easily scaled to meet the requirements of grid-level storage.^9,14,15^

Redox flow batteries can be composed from a variety of redox-active materials.^7,8,13^ AORFBs are of particular interest because they can be made from low-cost, Earth-abundant and non-toxic materials.^1–4,16,17^ Alkaline anthraquinone/ferrocyanide batteries are a prime example of AORFB, which hold record capacity retention rates within the field.^1,2,17–20^ These batteries are now in the process of being commercialized, though they currently exist on a small scale.^14,17,18,20^ Two of the main processes limiting the economic viability of anthraquinone/ferrocyanide batteries are electrolyte degradation and crossover (*i*.*e*., unwanted transport of redox-active material through the membrane).^11,21–27^ Both the battery materials and charging protocols need to be carefully designed to minimize these degradation processes and improve battery lifetime.^12,23,24,27–29^

The 2,6-dihydroxyanthraquinone (2,6-DHAQ)/ferrocyanide battery was the first example of a anthraquinone/ferrocyanide battery (Scheme 1),^1^ and it has since been used as a model AORFB in numerous studies.^23–26,30,31^ In the pioneering work by Zhao *et al*., on-line NMR was used to provide the first direct evidence for the previously proposed two-step, single-electron electrochemistry of 2,6-DHAQ in this battery.^23^ Additionally, this work revealed that a 1.7 V cell voltage hold results in the electrochemical degradation of 2,6-DHAQ into anthrone and anthrol derivatives.^1,23,31^ Building on these studies, Jing *et al*. demonstrated that 2,6-DHAQ could be regenerated from these electrochemical degradation products using a deep discharge to -0.2 V.^24^ By implementing this regeneration step, capacity fade rates (*i*.*e*., the rate of decline in maximum energy storage capacity) can be vastly reduced from 6.45% per day to 0.38% per day.^24^ Importantly, these pivotal studies were supported by on-line NMR analysis, highlighting the importance of this technique to the advancement of redox flow batteries.^23,24,26^

**Scheme 1:**
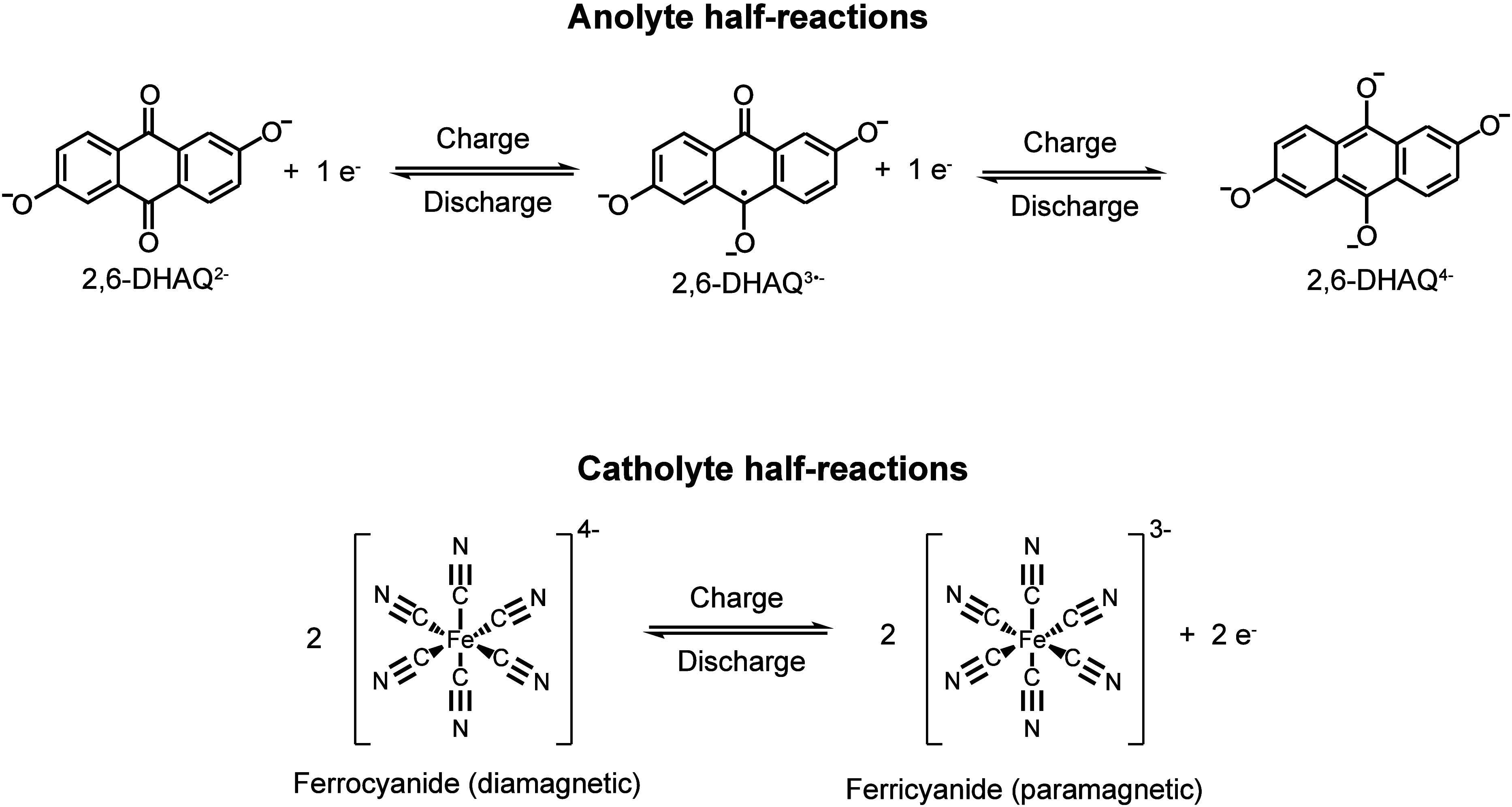
Redox Reactions Utilized in the Alkaline 2,6-DHAQ/Ferrocyanide Redox Flow Battery

Advances in material design have also contributed to improved battery lifetimes.^4,18,31^ More stable anthraquinone anolytes have been designed, such as 2,6-di­(3-phosphenopropyloxy)­anthraquinone (2,6-DPPAQ), which has a capacity fade rate of <0.014% per day.^18^ In 2,6-DPPAQ, phosphonate functional groups replace the hydroxyl functional groups of 2,6-DHAQ, affording both enhanced solubility and chemical stability.^1,18,32^ However, the impact of electrolyte crossover in operating redox flow batteries remains relatively unexplored.^30^ Notably, the impact of crossover on electrolyte stability has not yet been directly measured in any system. This is of particular importance in anthraquinone/ferrocyanide batteries, where the stability of the alkaline ferrocyanide/ferricyanide electrolyte is already under debate.^33–36^ Though significant research has been carried out to develop compatible organic catholytes, it is an ongoing challenge in the field, and ferrocyanide catholytes remain heavily relied on.^3,33,37–39^ As anolyte design progresses, the lifetime of these batteries becomes increasingly dependent on the impacts of crossover and ferrocyanide stability.

An important advantage of using on-line NMR to study electrolyte crossover and degradation is that the impacts of battery state-of-charge can be investigated.^23,30^ Indeed, the battery state-of-charge can be tracked by measuring the change of the solvent chemical shift.^30,40,41^ As ferricyanide (*i*.*e*., the charged form of ferrocyanide) is paramagnetic, the bulk magnetic susceptibility of the solution depends on its concentration in solution.^40–42^ The concentration of ferricyanide can then be calculated from changes in solvent chemical shift using the well-established Evans’ method.^40,41^ Given that the concentration of 2,6-DHAQ can also be measured, from the integral of its ^1^H NMR resonance, this gives on-line NMR the unique advantage of simultaneous ferricyanide and 2,6-DHAQ quantification.^30^ In this work, we exploit this advantage to investigate the impact of crossover on battery degradation at different stages of charge. We find that anolyte crossover causes side reactions in ferrocyanide electrolytes at high state-of-charge, evidenced by losses in ferricyanide and anolyte concentration, as measured by ^1^H NMR. These processes are highly dependent on the composition of the anolyte and can be mitigated by avoiding the use of hydroxyl-functionalized anolytes. The results presented herein suggest that the impact of crossover in anthraquinone/ferrocyanide batteries may have been underestimated in the AORFB field.

## Results

### Initial Observation of Crossover-Driven Degradation in Full-Cell Experiments

The impact of crossover on electrolyte degradation was investigated in the 2,6-DHAQ/ferrocyanide redox flow battery using the same on-line ^1^H NMR crossover detection method we developed previously (Figure 1, Experimental Methods 1 and 2).^30^ The NMR acquisition parameters and electrolyte flow rate were carefully optimized so that the resulting ^1^H NMR spectra were quantitative for 2,6-DHAQ (Experimental Methods 2 and Experimental Methods S1 and S2). Both the anolyte (0.1 M 2,6-DHAQ) and catholyte (0.25 M ferrocyanide with 0.04 M ferricyanide) were prepared in 1 M KOH in D_2_O, and the redox flow cell was equipped with a Nafion 211 membrane. The on-line ^1^H NMR apparatus was connected to the catholyte side (Figure 1) so that 2,6-DHAQ crossover and any subsequent side reactions could be observed. In our previous work, 2,6-DHAQ crossover rates were found to decrease over time when at high state of charge.^30^ One possible explanation for this was that some of the 2,6-DHAQ material was undergoing side reactions following its crossover into the catholyte. To further investigate this observation here, we applied a 1.5 V cell voltage hold to keep the battery at a high state-of-charge whilst looking for further evidence of side reactions (Experimental Methods 2).

**Figure 1:**
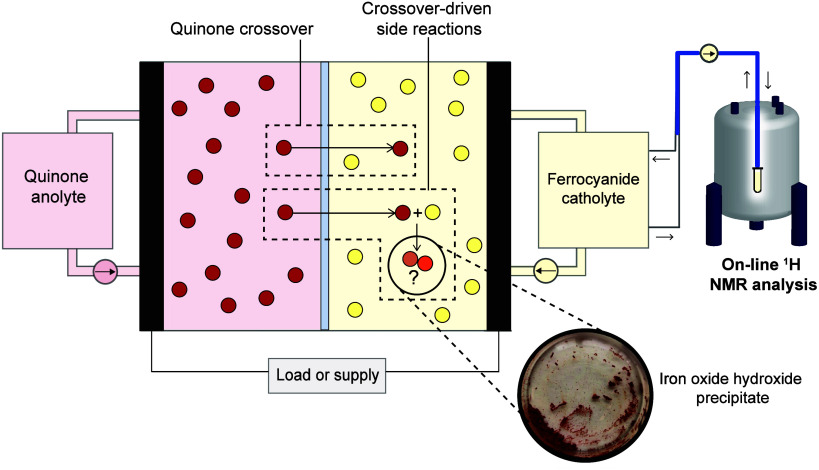
On-line NMR setup for characterizing crossover in a 2,6-DHAQ/ferrocyanide full-cell experiment. Crossover and crossover-driven side reactions are depicted with 2,6-DHAQ ions (represented as red spheres), ferrocyanide/ferricyanide ions (represented as yellow spheres) and the resultant degradation products (represented as orange-brown spheres). The inset photo shows iron oxide hydroxide formed in the ferricyanide catholyte.

In the first part of the experiment, 2,6-DHAQ accumulates in the catholyte during the 12 h rest period with a crossover rate of (4.2 ± 0.3) 10^–4^ mol m^–2^ h^–1^, consistent with our previous work (Figure 2a,b and Figure S1–S5).^30^ However, during the 1.5 V voltage hold step, the measured 2,6-DHAQ concentration in the catholyte starts to decrease, ultimately leading to a complete loss of 2,6-DHAQ ^1^H NMR signal (Figure 2a,b, Figure S1, and Figure S6–S8). Interestingly, the loss in 2,6-DHAQ signal does not correspond with the start of the voltage hold. Instead, it occurs just before ferricyanide reaches its maximum concentration, as measured by the state-of-charge (Figure 2b). Once the battery was discharged and allowed to rest, 2,6-DHAQ began to accumulate again in the battery catholyte due to crossover continuing. Notably though, the crossover rate of 2,6-DHAQ was lower at just (2.6 ± 0.1) 10^–4^ mol m^–2^ h^–1^ (Figure 2a,b, Figure S1 and Figure S9–S10). Furthermore, on disassembling the cell after experiments, an orange-brown precipitate was observed within the apparatus (Figure 1 and Figure S11). These results were the first indicators of the crossover-driven side reactions reported herein. As hypothesized, losses in 2,6-DHAQ ^1^H NMR signal result from the battery being held at a high state-of-charge. The next challenge was to identify which factors were important in causing this process to occur.

**Figure 2:**
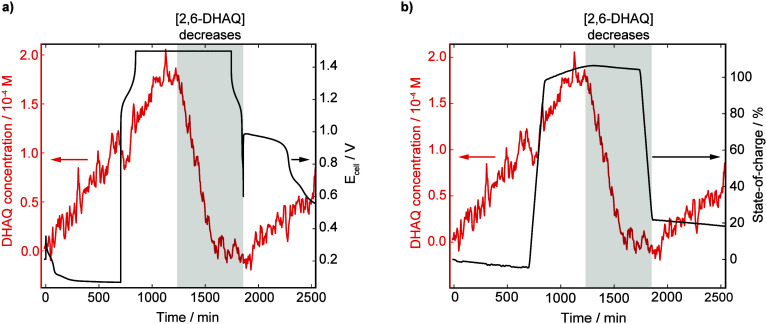
Plots showing 2,6-DHAQ crossover measured during the on-line ^1^H NMR full-cell study. The 2,6-DHAQ/ferrocyanide battery was allowed to rest for 12 h, followed by one charge–discharge cycle, including a 15 h voltage hold at 1.5 V. a) 2,6-DHAQ concentration measured in the catholyte (red trace) correlated with cell voltage (black trace). b) 2,6-DHAQ concentration measured in the catholyte (red trace) correlated with battery state-of-charge (black trace). The grey shaded area highlights the decrease in 2,6-DHAQ concentration in the catholyte.

### Simulated-Crossover Experiments

Within an operating battery there are many possible contributions to decreases in quinone or ferricyanide concentration, including: transport through the membrane (*i*.*e*., crossover),^11,27,30,18,43^ electrochemical conversion,^23–26^ chemical conversion^18,44,45^ and deposition.^1,3,38,46^ Therefore, to minimize the number of variables, the interactions between the catholyte and anolyte were studied outside of the battery environment, using a new ‘simulated-crossover’ methodology (Experimental Methods 3 and 4).

In a simulated-crossover test, the catholyte is spiked with a small volume of anolyte in an isolated vessel, mimicking crossover (Figure 3a). Quantitative ^1^H NMR is then used to monitor the changes in the organic species. Recalling that ferricyanide is paramagnetic and alters the bulk magnetic susceptibility of the solution, its concentration can be indirectly measured from the chemical shift of water (Experimental Methods 2, Experimental Methods S3 and Figure S12).^40,41^ This technique therefore enables the simultaneous quantification of aqueous quinone and ferricyanide following simulated-crossover.^30^

**Figure 3:**
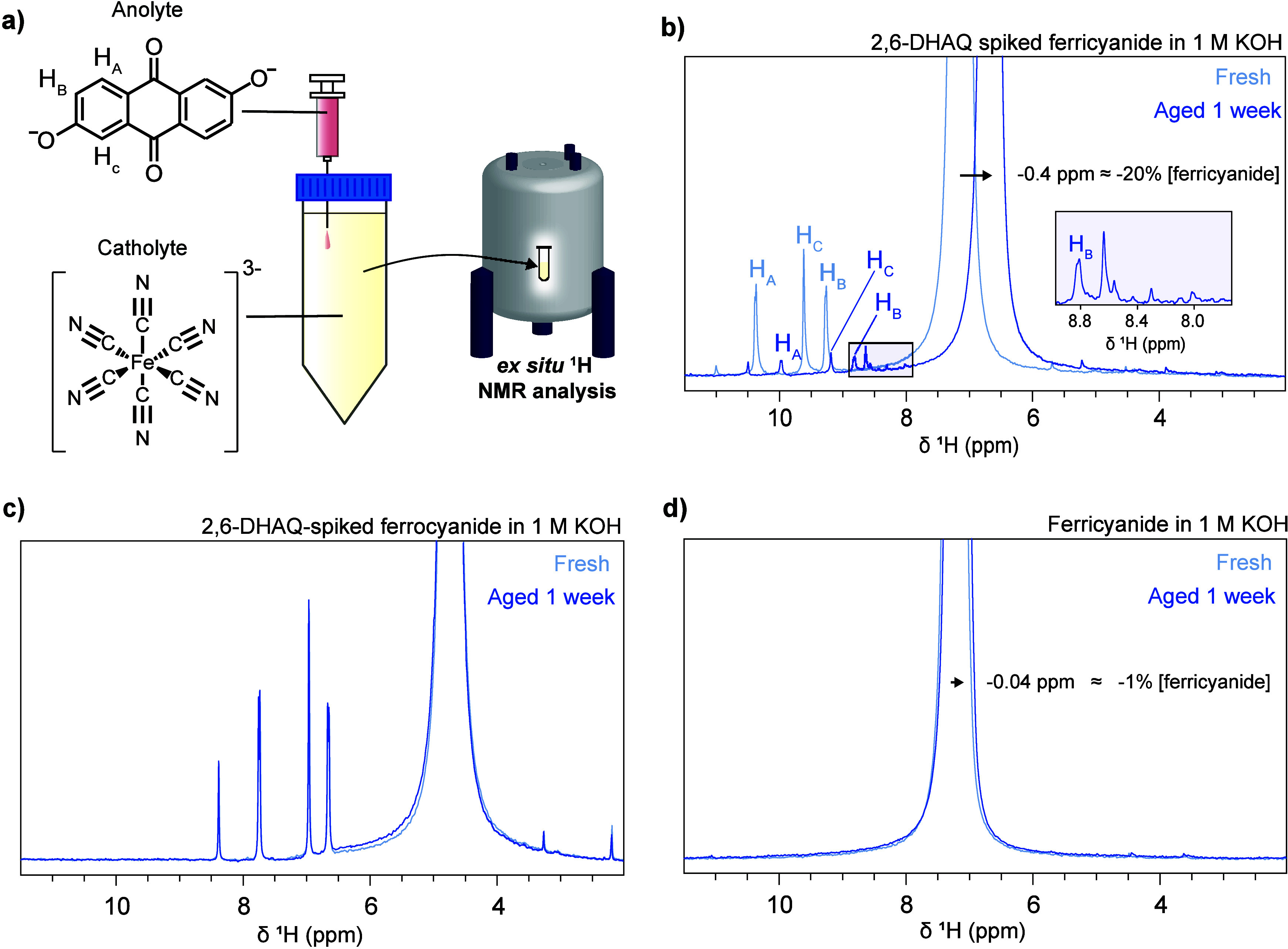
*Ex situ* ^1^H NMR simulated-crossover experiments. a) Schematic of an *ex situ* ^1^H NMR simulated-crossover experiment. b) ^1^H NMR spectra of 2,6-DHAQ-spiked ferricyanide catholyte before and after aging, where the shaded box highlights the new peaks that were observed next to resonance H_B_ in the aged catholyte. c) ^1^H NMR spectra of 2,6-DHAQ-spiked ferrocyanide catholyte before and after aging. d) ^1^H NMR spectra of ferricyanide catholyte before and after aging.

Simulated-crossover experiments were initially carried out on 0.29 M ferricyanide (*i*.*e*., charged catholyte, Fe­(III) oxidation state), as the drops in 2,6-DHAQ ^1^H NMR signal were observed at high-state-of-charge, where ferricyanide predominates over ferrocyanide (Figure 2a,b). A 5 mL portion of the ferricyanide catholyte was spiked with 0.1 mL of 0.1 M 2,6-DHAQ solution, so that the final concentration of 2,6-DHAQ was within the range measured in our previous on-line NMR crossover studies (∼2 mM).^30^ Both the anolyte and catholyte were prepared in 1 M KOH, same as in the full-cell experiments presented in the previous section.

Quantitative *ex situ* ^1^H NMR spectra were obtained for each solution within 1 h of preparation, and again 1 week later (Figure 3b). Remarkably, an (84 ± 12)% decrease in 2,6-DHAQ and (20.1 ± 0.3)% decrease in ferricyanide concentration was observed after 1 week (Figure 3b). For comparison, no significant change in 2,6-DHAQ concentration was measured in the 2,6-DHAQ-spiked ferrocyanide electrolyte (*i*.*e*., discharged catholyte, Fe­(II) oxidation state) after 1 week of aging (Figure 3c). Importantly, in a control experiment where a solution of 0.29 M ferricyanide was aged *in the absence of 2*,*6-DHAQ*, ferricyanide losses were minimal, at just (0.7 ± 0.2)% (Figure 3d).

On aging of the 2,6-DHAQ-spiked ferricyanide, the solution changed colour from red-orange to yellow, which is close to the original colour of ferricyanide before 2,6-DHAQ spiking (Figure S13). Additionally, in the aged solutions, orange-brown precipitates were observed, similar to those seen in the full-cell experiments (Figure S11). This is in contrast to the 2,6-DHAQ simulated-crossover experiments performed in ferrocyanide, where no colour changes or precipitate was observed on aging (Figure S13). Overall, the results from these simulated-crossover experiments mimic the effects seen in the full-cell studies, where losses in 2,6-DHAQ and ferricyanide are seen concurrently at high state-of-charge (Figure 2b, grey shaded box). As these losses in ferricyanide and 2,6-DHAQ are also detected in the simulated-crossover experiments, in the absence of an applied voltage, this indicates that the two components are reacting chemically.

In the aged sample of 2,6-DHAQ-spiked ferricyanide, a set of new peaks is also observed to the right of the 2,6-DHAQ H_B_ resonance (Figure 3b, shaded box). The appearance of these new peaks is another indicator that side reactions have taken place between ferricyanide and 2,6-DHAQ. Total correlation spectroscopy (TOCSY) was used to determine if these new proton environments were coupled to the 2,6-DHAQ protons (Experimental Methods 5). TOCSY uses through-bond interactions to reveal cross-peaks between all protons in a given spin system, provided there are couplings between every intervening proton.^47^ No cross-peaks were observed between the new peaks and the 2,6-DHAQ protons (Figure S14); however, diffusion-ordered NMR spectroscopy (DOSY) experiments, revealed that the diffusion coefficient of the new proton environment at 8.6 ppm (see inset, Figure 3b) matches that of 2,6-DHAQ (Experimental Methods 6 and Figure S15). This indicates that the new peak corresponds to a species with the same hydrodynamic radius as 2,6-DHAQ, or a species that is strongly interacting with 2,6-DHAQ (Figure S15).^48–50^

To gain further insight into how 2,6-DHAQ influences ferricyanide losses, on-line ^1^H NMR simulated-crossover experiments were performed (Figure 4a and Experimental Methods 4). Here, a flow NMR tube was used so that a quantitative ^1^H NMR spectrum of the catholyte could be acquired every 5 min throughout the simulated-crossover experiment (Experimental Methods 4, Experimental Methods S1 and Figure 4b,c). First, the ferricyanide loss rate was measured in the absence of 2,6-DHAQ. The measured ferricyanide loss rate was constant at 6 x 10^–4^ mol dm^–3^ h^–1^, which was consistent between repeats when performed in D_2_O and H_2_O (Figure 4c and Figure S16). Notably, this ferricyanide loss rate is higher than the ferricyanide loss rate measured in the *ex situ* NMR simulated-crossover experiments (1.3 x 10^–5^ mol dm^–3^ h^–1^), possibly reflecting the influence of sample flow or the shape of the reaction vessel on reaction rate (Experimental Methods 3 and 4).

**Figure 4:**
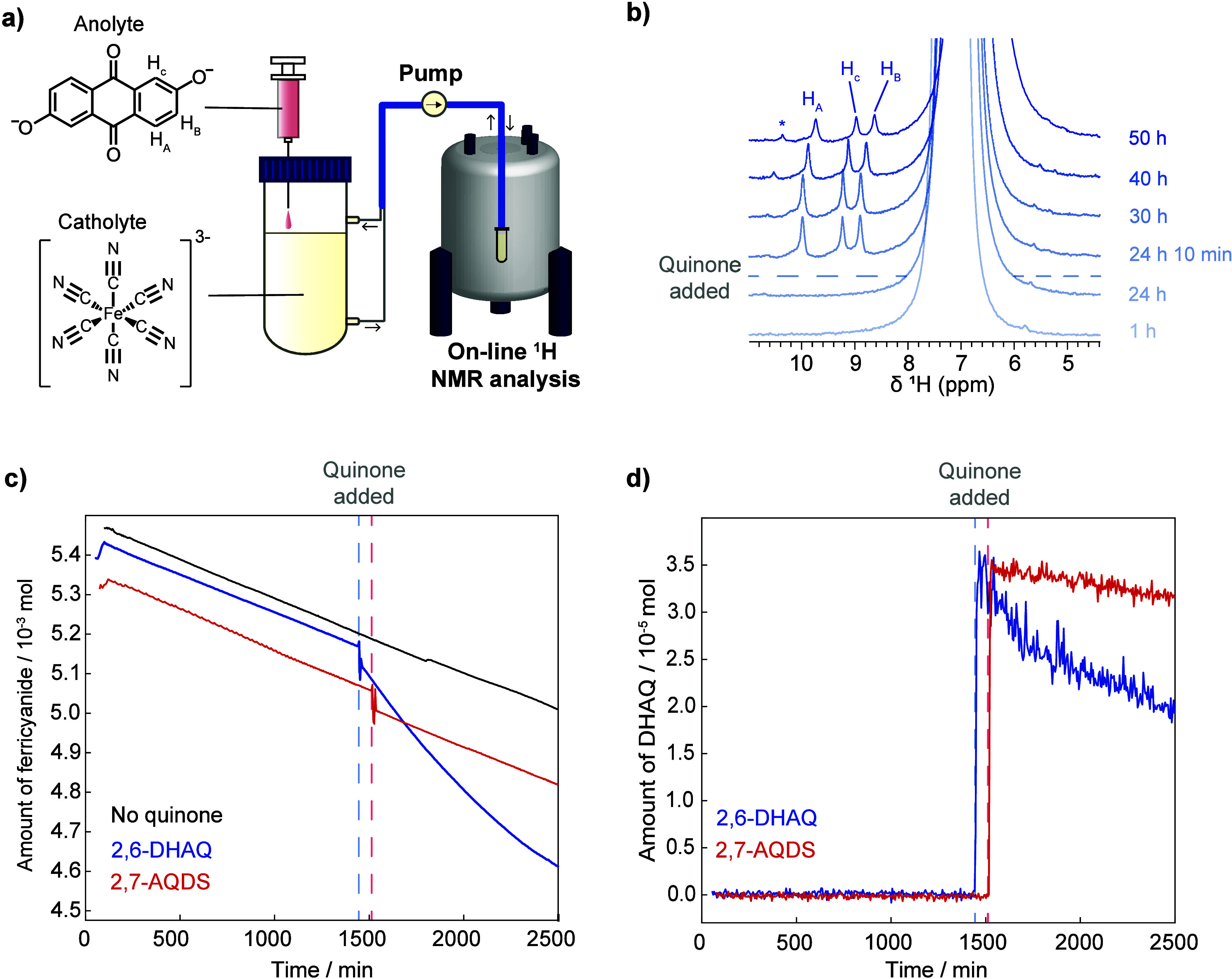
On-line simulated-crossover experiments. a) Schematic of the on-line ^1^H NMR simulated-crossover experiment. b) Selected ^1^H NMR spectra of ferricyanide catholyte during a 2,6-DHAQ simulated-crossover experiment, where the hypothesized formamide peak is highlighted with an asterisk. c) Measured changes in amount of aqueous ferricyanide in the whole catholyte during simulated-crossover experiments, in the absence of quinone (black trace), with 2,6-DHAQ spiking (blue trace) and 2,7-AQDS spiking (red trace). d) Measured changes in the amount of quinone in the whole catholyte during simulated-crossover experiments with 2,6-DHAQ (blue trace) and 2,7-AQDS (red trace). The blue and red dashed lines in c) and d) indicate the point at which 2,6-DHAQ and 2,7-AQDS were added, respectively.

After measuring background ferricyanide losses for 24 h, the ferricyanide catholyte was spiked with ∼2 mM 2,6-DHAQ anolyte. Strikingly, once 2,6-DHAQ was added (dashed line, Figure 4c), there is a small initial drop in the amount of ferricyanide followed by a three-fold increase in the ferricyanide loss rate to 1.8 x 10^–3^ mol dm^–3^ h^–1^. Control experiments were conducted where the catholyte mixture was spiked with the same volume of 1 M KOH (*i*.*e*., with no quinone present) and no drop in ferricyanide or increase in loss rate was observed (Figure S17). Intriguingly, the ferricyanide loss rate measured after the addition of 2,6-DHAQ is lower when performed in H_2_O (1.3 x 10^–3^ mol dm^–3^ h^–1^) instead of D_2_O (Figure S16). This is in contrast to ferricyanide losses measured in the absence of 2,6-DHAQ, which were the same in D_2_O and H_2_O (Figure S16). The lower ferricyanide loss rates observed in H_2_O relative to D_2_O could be explained as an inverse kinetic isotope effect, indicating that O–H bond breaking or formation is now involved in the rate-determining step.^51,52^ As this effect was only observed after the addition of 2,6-DHAQ, it indicates that a new ferricyanide loss mechanism is involved when 2,6-DHAQ is present.

Similar to the *ex situ* simulated-crossover experiments, losses in 2,6-DHAQ were also observed after its addition to ferricyanide (Figure 4b,d). Interestingly, the measured 2,6-DHAQ loss rate, (3.33 ± 0.08) x 10^–5^ mol dm^–3^ h^–1^, was nearly two orders of magnitude slower than the loss rate of ferricyanide, suggesting that ferricyanide losses are occurring via a catalytic process involving 2,6-DHAQ. To further test this hypothesis, *ex situ* simulated-crossover experiments were repeated at different anolyte:catholyte mixing ratios, revealing that 2,6-DHAQ and ferricyanide losses were both maximized at a ∼1:20 molar ratio (Figure S18).

Another interesting observation was the appearance of a new singlet peak in the ^1^H NMR spectrum of the ferricyanide catholyte after the addition of 2,6-DHAQ (Figure 4b, asterisk), which we assign as formamide. This assignment was based on the chemical shift difference of this peak relative to water and control experiments in samples where a formamide standard was intentionally added (Experimental Methods 6, Figure S19).^25^ Notably, formamide was also observed in the ^1^H NMR spectra of freshly prepared 2,6-DHAQ anolyte and ferrocyanide catholytes (Figure S20), and in *1 week aged samples* of ferricyanide catholyte (Figure S21). Formamide is not found in fresh samples of ferricyanide, and a key new observation from the on-line simulated-crossover experiment is that the addition of 2,6-DHAQ appeared to initiate the formation of formamide in the catholyte (Figure 4b). It is important to note that, although 2,6-DHAQ anolytes contain some formamide impurities (Figure S20),^25^ the rate at which it appeared in the system was significantly slower than that of 2,6-DHAQ mixing (Figure S22). This shows that formamide is being formed by a chemical reaction within the system, indicating a possible relationship between the formation of formamide and the loss of ferricyanide. As cyanide is the only nitrogen-containing component in the catholyte and anolyte, it is most likely being formed as an intermediate product of cyanide decomposition.^53–56^

To gain further insight into the mechanism of these crossover-driven side reactions, the same on-line ^1^H NMR simulated-crossover experiment was carried out with another common AORFB anolyte, anthraquinone-2,7-disulfonic acid (2,7-AQDS) in 1 M KOH. Excitingly, though an initial drop in ferricyanide was observed, the addition of 2,7-AQDS did not result in a significant increase in ferricyanide loss rate (Figure 4c). Similarly, 2,7-AQDS appeared to be much more stable in ferricyanide and was lost at a rate of (8.8 ± 0.6) x 10^–6^ mol dm^–3^ h^–1^, which is nearly four times slower than 2,6-DHAQ (Figure 4d). This important result shows that the crossover-driven side reactions reported herein can be mitigated by changing the anolyte structure.

### Anolyte Effects on Crossover-Driven Degradation

Motivated by the promising result that the observed side reactions are less severe in the presence of 2,7-AQDS compared to 2,6-DHAQ, the influence of anolyte structure on these reactions was investigated further. A series of *ex situ* simulated-crossover experiments were used to test the performance of various anolytes at the current forefront of the AORFB field (Experimental Methods 3, Figure S23–S26 and Table 1). In addition to 2,6-DHAQ and 2,7-AQDS, the high-performing anthraquinone 2,6-DPPAQ was tested (Experimental Methods 3 and Experimental Methods 7).^18^ Each anolyte tested had a different solubilizing functional group; 2,6-DHAQ possesses hydroxyl groups, whereas 2,7-AQDS and 2,6-DPPAQ have sulfonate and phosphonate groups, respectively.

**Table 1:**
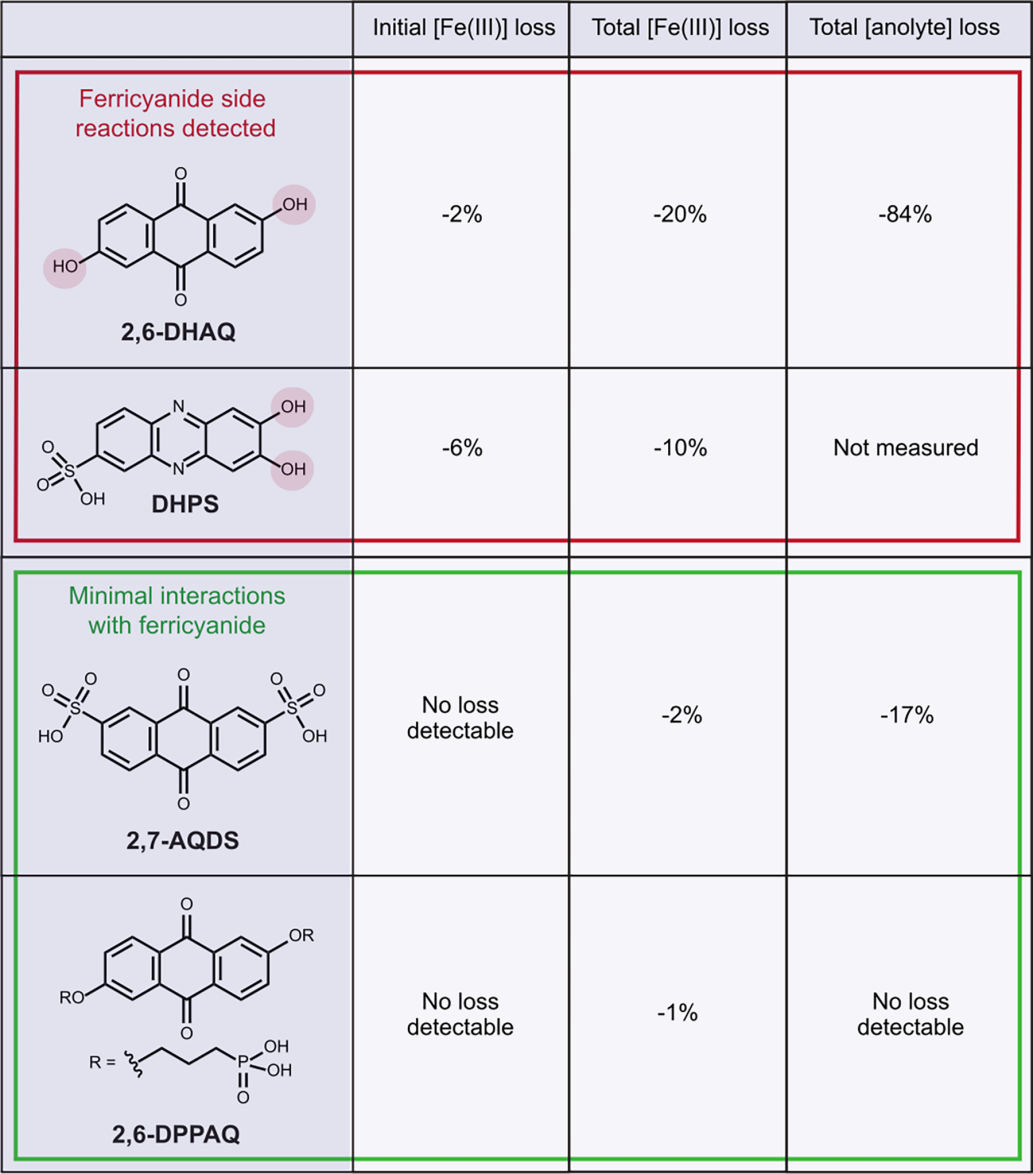
Comparison of the Performance of Different Anolytes in *Ex Situ* Simulated-Crossover Experiments (Experimental Methods 3)­^a^

a Showing skeletal formulas, initial percentage drop in ferricyanide concentration measured within 1 h of addition of the anolyte, and the total percentage drop in ferricyanide and quinone concentration after 1 week aging. Red and green boxes highlight anolytes where crossover-driven side reactions detected were significant (*i*.*e*., hydroxyl-functionalized anolytes) or minimal, respectively. Note that the change in DHPS concentration could not be quantified as the ^1^H NMR peaks were broad and overlapping and could not be accurately assigned (Figure S26).

Out of all the anthraquinone anolytes tested, the greatest ferricyanide and quinone losses were observed for 2,6-DHAQ. One of the possible explanations for this is that the hydroxyl groups in 2,6-DHAQ, which are deprotonated in alkaline conditions, and are able to coordinate with Fe^3+^. To further support this hypothesis, the two anolytes which do not have hydroxyl groups, 2,6-DPPAQ and 2,7-AQDS, displayed the lowest ferricyanide and anthraquinone losses (Table 1 and Table S1–S4). To determine if this phenomenon was exclusive to quinone-based anolytes, a third anolyte was tested; a high-performance phenazine anolyte with hydroxyl groups, 7,8-dihydroxyphenazine-2-sulfonic acid (DHPS, Table 1), once again prepared in 1 M KOH supporting electrolyte (Experimental Methods 3).^57^

The total ferricyanide losses observed with DHPS were comparable to those observed with 2,6-DHAQ after 1 week aging, showing that the side reactions observed are not exclusive to anthraquinones (Table 1 and Table S1–S2). The initial losses in ferricyanide (i.e., those measured 1 h after the addition of the anolyte) were higher than those observed with 2,6-DHAQ. This could be explained by the geometry of the hydroxyl groups in DHPS, which has hydroxyl groups in the *ortho* position relative to each other. Indeed, hydroxyl groups with this geometry are optimal for Fe^3+^ coordination, and this geometry is commonly seen in linker molecules for iron-quinone metal-organic frameworks.^58–61^ This suggests that the initial loss of ferricyanide is related to the ability of the anolyte to coordinate to Fe^3+^. Interestingly, however, the total ferricyanide loss after 1 week was still greater in the presence of 2,6-DHAQ than DHPS (Table 1, Table S1–S2). This could suggest that although the initial losses are related to coordination strength between anolyte and Fe^3+^, the losses are not all caused by direct coordination, and there is another underlying mechanism that causes ferricyanide losses to occur over longer time periods.

Another important observation was that orange-brown precipitates were observed in all the simulated-crossover experiments when ferricyanide was present, even in the absence of quinone (Figure S11). As these solids were also observed in the 2,6-DHAQ/ferrocyanide battery, which experienced 2,6-DHAQ signal losses, they appear to be relevant to the crossover-driven side reactions discussed above. Therefore, to better understand the formation of these solids, work was then carried out to determine their composition.

As a reminder of the context for this solid formation, the key results from each type of crossover experiment (see Experimental Methods 2-4 and Table S5) are summarized below:

- **On-line NMR 2,6-DHAQ/ferrocyanide full-cell experiments:** Losses in 2,6-DHAQ and ferricyanide are observed in ferrocyanide-based catholytes at high state-of-charge, when a 1.5 V voltage hold is applied to the redox flow cell.
- ***Ex situ*** **2,6-DHAQ simulated-crossover experiments:** Losses in 2,6-DHAQ and ferricyanide are also observed when the two components are mixed, in the absence of an applied voltage, indicating there is a chemical reaction between these two species.
- **On-line 2,6-DHAQ simulated-crossover experiments:** Background ferricyanide losses are observed in the absence of 2,6-DHAQ; however, the rate of ferricyanide loss was increased three-fold when 2,6-DHAQ is added. An inverse kinetic isotope effect was observed for ferricyanide losses, but only in the presence of 2,6-DHAQ. There is also evidence that formamide forms chemically within the system, which could be a product of cyanide ligand decomposition. Formamide formation was initiated when 2,6-DHAQ was added.
- **Impact of anolyte structure:** These crossover-driven side reactions were found to be mitigated when the anolyte did not have a hydroxyl group attached (*i*.*e*., minimal reactions were detected between ferricyanide and 2,6-DPPAQ or 2,7-AQDS). Side reactions were observed in phenazine and quinone anolytes containing hydroxyl groups.
- **Orange-brown precipitate was formed in all the experiments where ferricyanide was aged, even in the absence of added anolytes**.

### Investigation of Solid Formation

To facilitate the characterization of the orange-brown solids, they were prepared on a larger scale, using 500 mL of electrolyte of the same concentration as in the simulated-crossover experiments (Experimental Methods 8). The solids were prepared both in the presence and absence of 2,6-DHAQ (Experimental Methods 8), and the resulting solids were named FeQ-solids and Fe-solids, respectively (Figure S11). The first hypothesis was that these solids contained a mixture of iron (oxy) hydroxides, as this has previously been reported as a ferricyanide degradation product,^62,63^ and some organic impurities. The FeQ-solids and Fe-solids were therefore analyzed by Raman spectroscopy, which can be used to identify different iron minerals such as iron (oxy) hydroxides (Experimental Methods 9).^64–66^ The Raman spectra for the FeQ-solids and Fe-solids were very similar (Figure S27); both materials had significant peaks at ∼232 and ∼375 cm^–1^, and some weaker, broader peaks ∼505 cm^–1^ and ∼683 cm^–1^ (Figure S27). The Raman spectra of these solids did not match well with the spectra of pure iron­(III) oxide or iron­(III) hydroxide (Figure S27).^64–67^ However, comparing the Raman spectra of our FeQ-solids with those of iron oxide/hydroxide minerals on the RRUFF database (Figure 5a) revealed that they most closely resemble γ-FeOOH (a polymorph of iron oxide hydroxide, also known as Lepidocrocite).^68–70^

**Figure 5:**
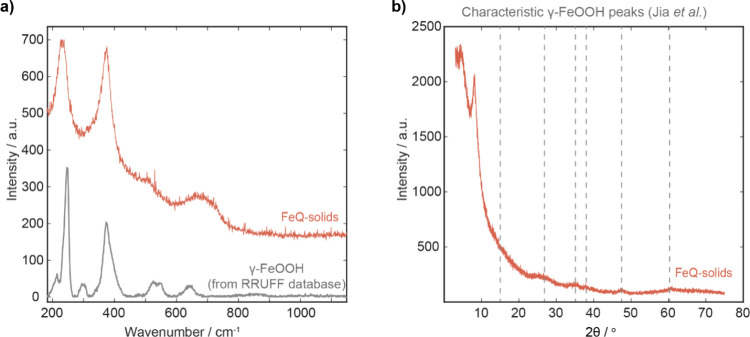
PXRD pattern and Raman spectra for the FeQ-solids compared to reference spectra for γ-FeOOH. a) Raman spectrum for FeQ-solids compared to reference spectrum of Lepidocrocite (γ-FeOOH) from the RRUFF database.^70^ b) PXRD pattern of FeQ-solids annotated with grey dashed lines indicating the key peaks from reference pattern of γ-FeOOH (Jia *et al*.^71^).

The FeQ-solids and Fe-solids were also analyzed using powder X-ray diffraction (PXRD) to characterize any crystalline components of the material (Experimental Methods 10). Both solids had PXRD patterns with few, broad peaks, further indicating the materials were mostly amorphous, or that they have a very small particle size (Figure 5b and Figure S28).^72,73^ However, some weak PXRD peaks were observed at 2θ of ∼26°, ∼34°, ∼38°, ∼47° and ∼61° (Figure 5b). Similar PXRD patterns have previously been assigned to γ-FeOOH particles, further supporting the hypothesis that this is a major component of the FeQ-solids.^71,74^ The FeQ-solid preparation was also repeated using a higher KOH concentration (1.2 M), to replicate possible concentration variations within the battery. The resulting material had strong PXRD peaks at 2θ of 18°, 21° and 26°, which are characteristic of the iron oxide hydroxide polymorph α-FeOOH, or Goethite (Figure S29).^64,75^

Combustion analysis and Inductively Coupled Plasma Mass Spectrometry (ICP-MS) showed that these solids were both composed of ∼50 wt % Fe, 2-6 wt % C and 1-2 wt % H and trace amounts of N (Experimental Methods 11). Both solids were prepared twice, and no significant difference was found in the elemental composition of the FeQ-solids and Fe-solids (Table S6). The expected composition of γ-FeOOH is around ∼63 wt % Fe, 1 wt % H and 36 wt % O. Therefore, elemental composition of the FeQ-solids and Fe-solids support the conclusion that these precipitates are composed of a mixture of iron oxide hydroxide and some organic impurities (Table S6).

Iron oxides/hydroxides are also known to be magnetic materials;^76–78^ therefore, the FeQ-solids and Fe-solids were also analyzed using vibrating sample magnetometry (VSM, see Experimental Methods 12). These measurements confirmed that both solids have a magnetic moment, which was measured at 0.5-0.005 emu/g and 0.7-0.6 emu/g for the FeQ-solids and Fe-solids, respectively (Figure S30). The magnetic moment of both materials was within the range expected for γ-FeOOH.^76^ The lower magnetic moment measured for FeQ-solids relative to Fe-solids could indicate there is additional non-magnetic material in the FeQ-solids. To investigate the possibility of the FeQ-solids containing some coordinated 2,6-DHAQ, they were dissolved in deuterium chloride in D_2_O and analyzed by ^1^H NMR (Experimental Methods 13). Though there was evidence of some organic impurities such as formamide, no evidence of 2,6-DHAQ was found (Figure S31). Considering 0.1 mM 2,6-DHAQ was detectable using the same NMR experiment, the 2,6-DHAQ content must be below 0.8% of the mass of the FeQ-solids (Experimental Methods 13). It should be noted that 2,6-DHAQ has low solubility in acids, however, the FeQ-solids were not soluble in KOH. These results suggest that if 2,6-DHAQ is incorporated in the precipitate formed in the 2,6-DHAQ/ferrocyanide battery, it is not a significant component.

Summarizing, Raman, PXRD, VSM, and elemental analysis revealed that the FeQ-solids and Fe-solids have similar composition, likely amorphous γ-FeOOH. The magnetic moment of the FeQ-solids was lower than that of Fe-solids, which could indicate the FeQ-solids contain more non-magnetic material. We note that, as these magnetic precipitates settled to the bottom of the NMR tube (i.e., outside the detection region), they would not be expected to impact the accuracy of the ferricyanide concentration measured (Experimental Methods S3). Solution-state NMR of the dissolved FeQ-solids did not provide evidence of incorporated 2,6-DHAQ; however, other organic impurities such as formamide were detected. The evidence therefore suggests that both the FeQ-solids and Fe-solids are composed of a mixture of amorphous iron oxide hydroxide with a small amount of incorporated organic material.

## Discussion

We finally discuss the possible mechanisms through which anolyte crossover may exacerbate ferricyanide losses. First, it is important to note that ferricyanide is known to degrade in alkaline conditions.^34,35,62,63,79,80^ The rate of this degradation is greater at higher electrolyte pH^35,62^ and in the presence of high frequency visible light and UV light.^62,63,80,81^ This is because light at these wavelengths promote the loss of cyanide ligands, which are replaced by water and then hydroxide ions.^62,63,80^ In this process, ferricyanide is irreversibly converted to a mixture of insoluble iron oxide and hydroxide precipitates over time.^62,63,80,82^ However, the exact mechanisms for capacity losses in ferrocyanide-based batteries are still debated;^33–36^ Fell *et al*. recently defended the use to ferrocyanide electrolytes in AORFBs, claiming they are stable in ambient light.^33^ Their argument is that the loss of ferricyanide is due to a chemical reduction to ferrocyanide, driven by a coupled chemical oxygen evolution reaction (OER). In this case, ferricyanide can be regenerated, which would not be the case if it were converted to insoluble iron oxide/hydroxide precipitates.

In this report, evidence for ferricyanide losses were measured from the changes in bulk magnetism of the catholyte using ^1^H NMR. No kinetic isotope effect was observed for background ferricyanide losses in the absence of 2,6-DHAQ; this is consistent with commonly proposed ferricyanide degradation mechanisms, where the rate-determining step involves the dissociation CN^–^ ligands, and does not involve O–H bond breakage.^35,63,79,80,83^ Furthermore, orange-brown precipitates were also observed to form from the ferricyanide electrolyte, which we have shown are likely composed of a mixture of amorphous iron oxide hydroxide with a small amount of incorporated organic material.

The key new observation of our study is that ferricyanide losses were exacerbated by both phenazine and quinone anolytes possessing hydroxyl groups. The two anolytes that did not possess strong coordinating groups, 2,7-AQDS and 2,6-DPPAQ, were found to have minimal interactions with ferricyanide. These results strongly suggest that Fe­(III)-anolyte coordination is an important step in the crossover-driven side reactions observed. It must be noted, however, that the measured rate of ferricyanide loss was nearly two orders of magnitude higher than the rate of 2,6-DHAQ loss. Furthermore, losses of ferricyanide and 2,6-DHAQ still cannot be explained fully by the small quantity of solid precipitates obtained, which was just ∼18 mg from a 500 mL solution containing 2402 mg of 2,6-DHAQ (Experimental Methods 8). To explain the full 80% loss of 2,6-DHAQ, the solid precipitates would need to contain 1922 mg of 2,6-DHAQ. Characterisation of these precipitates also indicated that 2,6-DHAQ is not a major component of these solids.

We therefore conclude that anolyte-Fe^3+^ coordination is contributing to electrolyte degradation indirectly, and we propose that coordination could catalyse the reactions that lead to ferricyanide decomposition. Recalling that ferricyanide is known to degrade in alkaline conditions via the reversible loss of cyanide ligands, any process that removes these cyanide ligands could therefore be expected to increase the rate of ferricyanide degradation.^35,63,79,80,83^ In our on-line simulated-crossover experiments, formamide was found to form after the addition of 2,6-DHAQ to ferricyanide, which is a known intermediate product of cyanide decomposition in aqueous environments.^53,55,56^ The on-line simulated-crossover results indicate that 2,6-DHAQ increases the rate of formamide formation in ferricyanide, suggesting that 2,6-DHAQ could be catalysing the dissociation and decomposition of the cyanide ligands. Interestingly, when 2,6-DHAQ is added, there is also evidence that the rate-determining step in ferricyanide removal is changing. In the absence of 2,6-DHAQ, ferricyanide losses occur at the same rate in D_2_O and in H_2_O. However, in the presence of 2,6-DHAQ, an inverse kinetic isotope effect was observed, indicating that the rate-determining step for ferricyanide removal involves the breaking or formation of an O–H bond.^51,52^ Together, this supports the conclusion that in the presence of 2,6-DHAQ, there is an additional mechanism of ferricyanide loss, likely operating through anolyte-Fe^3+^ coordination and the subsequent decomposition of cyanide ligands (Scheme S1).

## Conclusions

Concluding, in this work we have presented the first evidence of crossover-driven side reactions in aqueous organic redox flow batteries. These side reactions increase the rate of degradation of ferrocyanide catholytes via interactions between ferricyanide and anolyte material. Furthermore, simulated-crossover experiments have been developed, which can be used to study these side reactions outside of the battery environment. Challenges in developing suitable organic catholytes^16,38^ mean that ferrocyanide is still heavily relied on in the field.^33^ Though these batteries are currently used on a small scale in industry,^20^ their long-term stability needs to be established before they are suitable for large-scale grid-level energy storage. The evidence presented in this report suggests that the anolyte chosen significantly influences the stability of the ferrocyanide electrolyte, and hence the lifetime of the battery, and our work therefore reveals new important design rules for anolyte development. It is also possible that similar processes could occur in similar organometallic catholytes, which are emerging as promising replacement for ferrocyanide.^39,84,85^

Furthermore, our work also shows that standard *ex situ* measurements could lead to an underestimation of crossover in ferrocyanide-based batteries if these crossover-driven side reactions are ignored. Our simple *ex situ* NMR simulated-crossover experiments can identify when these side reactions are occurring, so that unfavorable anolyte-catholyte combinations can be avoided. These insights not only enable more accurate crossover quantification but will also assist the development of longer-lasting ferrocyanide-based redox flow batteries.

## Experimental Section

### Experimental Methods 1 : Materials

The organic redox-active material used in the anolyte was 2,6-dihydroxyanthraquinone (2,6-DHAQ, >98% purity, AK Scientific). The inorganic redox-active components used in the catholyte were potassium ferrocyanide trihydrate and potassium ferricyanide (both 99%+ purity, ACROS Organics). Unless otherwise stated in the main text, all electrolytes were prepared in deuterium oxide (D_2_O, 99.98% atom% D, Sigma-Aldrich), with 1 M potassium hydroxide as the supporting electrolyte (KOH, analytical reagent grade, 85% purity, Fisher Chemical). For the full-cell on-line NMR experiment, 22 mL anolyte (0.1 M 2,6-DHAQ) and 22 mL catholyte (0.25 M potassium ferrocyanide trihydrate with 0.04 M potassium ferricyanide) were used. The ion-selective membrane used was Nafion 211 (25 μm dry thickness, FuelCellStore). The Nafion membranes were pre-treated, using the method described by Zhao *et al*.^23^ Firstly, they were soaked in Milli-Q water at 80 ^o^C for 20 min. Following this, they were placed in 5 wt % H_2_O_2_ solution (prepared from 30 wt % H_2_O_2_ in H_2_O, Sigma-Aldrich) for 35 min. Finally, the membranes were rinsed in Milli-Q water and then stored in 0.1 M KOH solution for at least 24 h before use. Untreated carbon felt was used as the electrode material (SIGRACELL® battery felt, GFD4,6 EA, SGL Carbon). The anolytes used in the simulated-crossover experiments were 2,6-DHAQ, anthraquinone-2,7-disulfonic acid disodium salt (2,7-AQDS, 80% purity, Biosynth), 2,6-di­(3-phosphenopropyloxy)­anthraquinone (2,6-DPPAQ, synthesized by Anqi Wang, Imperial College London) and 7,8-Dihydroxyphenazine-2-sulfonic acid (DHPS, 95% purity, BLD Pharmatech GmbH). The iron­(III) oxide and iron­(III) hydroxide standards were both α-phase, purchased from Alfa Aesar. The iron­(III) oxide and iron­(III) hydroxide were 98% and 99%+ pure, respectively. The formamide standard was 99.5% pure, purchased from Acros Organics. Deuterium chloride (DCl, 35 wt % in D_2_O, ≥99 atom % D, Sigma-Aldrich) was used to dissolve the FeQ-solids for solution-state ^1^H NMR analysis.

### Experimental Methods 2 : Full-Cell On-Line NMR Crossover Experiment

The same redox-flow battery setup was used as described in our previous work.^30^ The electrochemical cell was a Scribner Associates Inc. commercial Redox Flow Cell Test Fixture, purchased from Alvatek Ltd., Romsey. The current collectors were gold-plated copper, two serpentine flow fields were graphite, the four gaskets were Viton (0.7 mm thick) and two flow frames were PTFE (0.080” thick). In the assembled flow cell, the gaskets were placed between the flow fields, PTFE flow frames and a Nafion 211 membrane. A carbon felt electrode (5 cm^2^, 4.6 mm thick) was placed in each PTFE flow frame. The flow cell was sealed with bolts and tightened to 2 N m using a torque wrench. Perfluoroalkoxy polymer inlet and outlet tubes (PFA, 1/8” OD, Swagelok) were inserted into the back of the flow fields, through the aluminum endplates and current collectors, and secured with Viton O-rings were used to prevent electrolyte leaks. The electrolytes were pumped through the redox-flow cell at a rate of 60 rpm (∼16 mL min^–1^) using peristaltic pumps (MasterFlex L/S Pump, 07528-10; Easy-load Pump Head, 77202-60; MasterFlex ChemDurance Pump Tubing, 06442-14, #14), purchased from Cole-Parmer Instrument Company Ltd., St Neots. The anolyte was 22 mL 0.1 M 2,6-DHAQ (prepared in 1 M KOH/D_2_O) and the catholyte was 22 mL of 0.25 M potassium ferrocyanide and 0.04 M potassium ferricyanide (prepared in 1 M KOH/D_2_O). The electrolytes were both degassed with nitrogen for 30 min before the nitrogen inlet and outlet needles were removed.

The on-line NMR apparatus and experiment protocol was the same as described in our previous work.^30^ A commercial NMR flow tube (InsightMR, Bruker), was equipped with 1/16” OD PFA transfer tubing and connected to the catholyte reservoir. A third peristaltic pump was used to supply the NMR spectrometer with catholyte at a flow rate of 6 rpm (∼1.6 mL min^–1^), which is within the quantitative flow rate regime for 2,6-DHAQ H_A_ (Experimental Methods S1 and S2).^30^ Once the NMR was setup, the pseudo-2d NMR and electrochemical data acquisition was started simultaneously (see Experimental Methods S1 for further details on the quantitative NMR protocol used). A portable potentiostat (BioLogic SP-150) was used for battery cycling, using a time interval of 1 s between data point collections. The charging protocol used in this experiment consisted of a 12 h rest period, followed by a 50 mA constant-current charge to 1.5 V, a 15 h voltage hold at 1.5 V, a 50 mA constant-current discharge to 0.6 V and a final 12 h rest period.

The ferricyanide concentrations were measured indirectly from the change in water ^1^H NMR chemical shift, as described in our previous work,^30^ inspired by the work by Zhao *et al*. (see Experimental Methods S3 and Figure S12).^40^ The 2,6-DHAQ concentration was determined by integrating 2,6-DHAQ proton resonance H_A_. The signal was calibrated by making a calibration curve for a series 2,6-DHAQ solutions of known concentration (Experimental Methods S2 and Figure S32).

The limit of detection for this method was calculated as the concentration at which at which the signal-to-noise ratio (SNR) would be 3:1. The limit of detection was estimated to be 0.03 mM, using the ratio of root-mean-square (RMS) of the noise to 2,6-DHAQ H_A_ signal for a calibration solution of 100 mM 2,6-DHAQ.

### Experimental Methods 3 : *Ex Situ* ^1^H NMR Simulated-Crossover Experiments

All electrolytes were prepared fresh and analysed by ^1^H NMR within 1 h of preparation to minimize variation caused by differing degrees of electrolyte degradation at the start of the experiment. All electrolytes were prepared in 1 M KOH in D_2_O. The anolyte concentration was 0.1 M and the catholyte was 0.29 M ferricyanide or ferrocyanide. These concentrations were chosen so that they were representative of the concentrations used in the redox-flow battery experiments. In a 15 mL centrifuge vial, the catholyte (5 mL) was spiked with the anolyte (0.1 mL). Note that the shape of the container in which the catholyte was contained was found to influence the results of the experiment, so this was kept consistent between all samples. All samples were stored in a drawer in sealed 15 mL centrifuge vials.

A sample of the spiked electrolyte was removed for quantitative ^1^H NMR analysis immediately after preparation and again after 1 week. The ^1^H NMR experiments were performed by direct excitation with a 90° pulse on a 400 MHz standard bore Bruker magnet equipped with a 5 mm HD Smart Probe and Bruker Avance III spectrometer. All spectra were referenced to the chemical shift 4.8 ppm using an external D_2_O reference and the spectrometer lock was disabled. The probe temperature was set to 25 ^o^C for all experiments. The 2,6-DHAQ and ferricyanide concentrations were measured as described in Experimental Methods S2 and S3, respectively.

### Experimental Methods 4 : On-Line ^1^H NMR Simulated-Crossover Experiments

As with the *ex situ* ^1^H NMR simulated-crossover experiments, all electrolytes were prepared fresh and analysed by ^1^H NMR within 1 h of preparation. The start of the experiment time was recorded as the time at which the catholyte was prepared. All electrolytes were prepared in 1 M KOH in D_2_O (except where specifically stated in the text), and the anolyte concentration was 0.1 M quinone (2,6-DHAQ or 2,7-AQDS) and the catholyte was 0.29 M ferricyanide. The on-line apparatus was set up using with a sealed glass burette reservoir connected to a flow NMR tube. The catholyte (20 mL) was added to the glass reservoir, which was then sealed. The outlet tap was opened at the bottom of the reservoir, and the catholyte was pumped through the flow NMR tube using a peristaltic pump set to 6 rpm (1.6 mL min^–1^). The catholyte was continuously analysed using quantitative ^1^H NMR as described in Experimental Methods 2 and Experimental Methods S1.

Once NMR acquisition had commenced, the electrolyte reservoir was sealed degassed with nitrogen for 30 min. A light was kept on in the room so that the light exposure was constant. The rate of ferricyanide loss was found to fluctuate when the light was not kept on, likely due to the variation in light throughout the day. After ∼12 h, the catholyte was then spiked with 0.4 mL of the 0.1 M quinone anolyte. The quinone anolyte was degassed for 30 min prior to its addition to the catholyte. The flow rate was increased to 20 rpm (∼5.3 mL min^–1^) for 15 min, to aid mixing within the system. The flow rate was then returned to normal (1.6 mL). For the control experiment, the same procedure was used, but the catholyte was spiked with the degassed 1 M KOH supporting electrolyte.

The ferricyanide and quinone loss rates were determined by performing a linear fit of the change in amount of ferricyanide or quinone (mol) over time (h). The concentration of ferricyanide and quinone was determined as described in Experimental Methods S1–S3. The linear fits were performed on MATLAB using polyfit, and 95% confidence limits were used as the error estimation for the permeability and crossover rate. The estimated 2,6-DHAQ and 2,7-AQDS loss rate were measured from 12 h of data, starting 15 min after their addition to the ferricyanide catholyte. The data from quinone addition to 15 min after was discarded from the loss rate measurement to ensure the measurement was taken once the system was fully mixed.

### Experimental Methods 5 : Total Correlation Spectroscopy (TOCSY)

TOCSY uses through-bond interactions to create correlations between all protons within a given spin system, where there are couplings between every intervening proton. Here the TOCSY measurements were performed on an aged sample of 2,6-DHAQ-spiked ferricyanide (with and without a formamide standard). Experiments were performed in a standard 5 mm solution-state NMR tube, using the mlevphpr.2 pulse sequence available on Bruker Topspin software. A mixing time of 80 ms was used with a spin lock strength of 4650 Hz and pre-saturation strength of 23 Hz. The spectra were taken on a 600 MHz standard bore Bruker magnet equipped with a 5 mm BBI solution-state probe and Bruker Neo spectrometer. The spectrometer was locked onto the HOD peak, and solvent suppression was applied to the HOD peak.

### Experimental Methods 6 : Diffusion Ordered NMR Spectroscopy (DOSY)

DOSY measurements were performed on an aged sample of 2,6-DHAQ-spiked ferricyanide in a standard 5 mm solution-state NMR tube, using the ledbpgp2s pulse sequence available on Bruker Topspin software. The diffusion time was set to 0.06 s and the gradient pulse length was 1200 μs. The spectra were taken on a 600 MHz standard bore Bruker magnet equipped with a 5 mm BBI solution-state probe and Bruker Avance Neo spectrometer. The spectrometer was locked onto HOD, and solvent suppression was applied to the HOD peak. The DOSY data was processed using Bruker Dynamics Centre software.

### Experimental Methods 7 : Synthesis of 2,6-DPPAQ

The synthesis procedure is adapted from the literature.^18^ A mixture of 2,6-dihydroxyanthraquinone (4.80 g, 20.0 mmol), diethyl (3-bromopropyl)­phosphonate (13.00 g, 50.2 mmol), anhydrous K_2_CO_3_ (11.0 g, 79.6 mmol) and DMF (100 mL) was heated to 100 °C for 18 h. The mixture was cooled and DMF was removed. The solid was thoroughly washed with water and extracted with DCM (100 mL, three times). The DCM solution was combined and dried over anhydrous MgSO_4_. Trimethylsilyl bromide (TMSBr, 30.60 g, 199.9 mmol) was added to the solution, which was stirred at 25 °C for 15 h. DCM and excess TMSBr were removed. The crude precipitate was washed thoroughly with water and dried under vacuum to give a yellow powder (9.20 g; yield, 95%). ^1^H NMR (500 MHz, DMSO-*d*_6_) δ (ppm) 8.10 (d, J = 8.6 Hz, 2H, ArH), 7.52 (d, J = 2.7 Hz, 2H, ArH), 7.37 (dd, J = 8.7, 2.7 Hz, 2H, ArH), 4.23 (t, J = 6.4 Hz, 4H), 1.97­(m, 4H), 1.71 (m, 4H); ^13^C NMR (126 MHz, DMSO-*d*_6_) δ (ppm) 181.1, 163.4, 135.2, 129.5, 126.4, 120.6, 110.6, 68.5, 68.3, 24.4, 23.4, 22.8, 22.7.

### Experimental Methods 8 : Preparation of Fe-Solids and FeQ-Solids

The FeQ-solids were prepared by mixing 10 mL of 0.1 M 2,6-DHAQ with 500 mL of 0.29 M potassium ferricyanide, both of which were prepared in 1 M KOH supporting electrolyte in H_2_O (except for one sample, which was prepared in 1.2 M KOH, as inidcated in the main text). The resulting solution was divided between two 250 mL Pyrex containers and left out in ambient light for over 1 month. The resulting solids were collected and washed with 3 x 50 mL deionized water, followed by 3 x 50 mL ethanol and finally 3 x 50 mL acetone. After removing excess acetone, the solids were dried using nitrogen, then placed in a vacuum oven set to 90 ^o^C for at least 24 h before analysis. The Fe-solids were prepared in the same way, using just 500 mL of 0.29 potassium ferricyanide, without any added 2,6-DHAQ.

### Experimental Methods 9 : Raman Spectroscopy

The Raman spectra were collected on a HORIBA Scientific LabRAM HR Evolution Raman Spectrometer equipped with a Syncerity OE detector and a 785 nm edge laser with a 750 nm filter. Laser power was reduced by filter to 1 mW. Spectra were collected between 100 and 2000 cm^–1^, using 10 accumulations each with an acquisition time of 100 s.

### Experimental Methods 10 : Powder X-ray Diffraction

The powder X-ray diffraction data was collected on a Malvern Panalytical Empyrean instrument, equipped with an X’celerator Scientific detector using non-monochromated Cu Kα radiation (λ = 1.5418 Å). Samples were placed on a zero-background silicon sample holder and measured in reflection geometry with sample spinning. The data was collected at room temperature over a 2θ range of 3 – 75 °, with an effective step size of 0.017 °. The total collection time for the FeQ-solids and Fe-solids prepared using 1 M KOH supporting electrolyte was 15 h. The total collection time for the FeQ-solids prepared using 1.2 M KOH supporting electrolyte was 5 h.

### Experimental Methods 11 : Elemental Analysis

Fe content was determined via inductively coupled plasma optical emission spectroscopy using a Thermo Scientific iCAP-7400 ICP spectrometer. C, H and N concentrations were determined via CHN combustion analysis using an Exeter Analytical CE-440, with combustion at 975 °C.

### Experimental Methods 12 : Vibrating Sample Magnetometry

The VSM measurements were performed on a LakeShore 8600 Series VSM at room temperature. The magnetic field was swept between 15000 and -15000 Oe.

### Experimental Methods 13 : Solution-State ^1^H NMR of Dissolved Fe- and FeQ-Solids

The FeQ-solids (2.7 mg) were dissolved in 0.1 mL of DCl, then diluted with a further 0.8 mL D_2_O and transferred into a standard 5 mm NMR tube. The ^1^H NMR spectrum of the dissolved FeQ-solids was collected using a 400 MHz standard bore Bruker magnet equipped with a 5 mm HD Smart Probe and Bruker Avance III spectrometer. A pulse-acquire experiment was used by implementing the ‘zg’ pulse sequence available on Bruker TopSpin software. The d1 was 3 s, and 128 scans were collected. The same experiment was repeated on a solution of 0.1 mM 2,6-DHAQ (prepared in 1 M KOH/D_2_O) to estimate the limit of detection. The spectrum was referenced at the HOD peak in the spectrum at 4.8 ppm.

## Supplementary Material



## Data Availability

All raw experimental data files and supporting code are available in the Cambridge Research Repository, Apollo, with the identifier: 10.17863/CAM.112904.

## Notes

Additional details for experimental methods, characteristic data and mechanistic scheme (Experimental Methods S1–S3, Figures S1–S32, Tables S1–S6 and Scheme S1) (PDF)
