# Supplementary material for: Revealing and Mitigating Crossover-Driven Side Reactions in Ferrocyanide-Based Redox Flow Batteries
Source: ACS Electrochem. 2025 Aug 14;1(10):2071–84. doi: 10.1021/acselectrochem.5c00178 (PMC12498410; doi:10.1021/acselectrochem.5c00178)
Supplement: Supplementary file 1 [file ec5c00178_si_001.pdf]

Supporting Information for: Revealing and Mitigating Crossover-Driven Side Reactions in Ferrocyanide-Based Redox Flow Batteries

Emma J. Latchem,[a] Thomas Kress,[a] Muireann Anna de h-Óra,[b] Anqi Wang,[c,d] Qilei Song[c] and Alexander C. Forse[a*]

[a]Yusuf Hamied Department of Chemistry, Lensfield Rd, University of Cambridge, CB2 1EW

[b]Department of Materials Science and Metallurgy, Charles Babbage Rd, University of Cambridge, CB3 0FS

[c]Barrer Centre, Department of Chemical Engineering, Imperial College London, SW7 2AZ

[d]Physical Science and Engineering Division, King Abdullah University of Science and Technology, Thuwal, Saudi Arabia

*afc50@cam.ac.uk

Table of Contents

Experimental Methods……………………………………………………………..

Experimental Methods S1 : Quantitative on-line NMR acquisitions parameters S6

Experimental Methods S2 : Calibration of 2,6-DHAQ concentration...................... S7

Experimental Methods S3 : Calibration of ferricyanide concentration S7-S8

Figures……………………………………………………………………………..

Figure S1 : Full-cell simulated-crossover data (Experimental Methods 2), with coloured spots and letters (A-I) indicating the timepoints used for the ^{1}H NMR spectra shown in Figures S2-S10... S9

Figure S2 : ^{1}H NMR spectrum of the catholyte from the start of the full-cell crossover experiment S9

Figure S3 : ^{1}H NMR spectrum of the catholyte from the middle of the first cell rest period during the full-cell crossover experiment.. S10

Figure S4 : ^{1}H NMR spectrum of the catholyte from the end of the first cell rest period during the full-cell crossover experiment... S10

Figure S5 : ^{1}H NMR spectrum of the catholyte from the end of the 50 mA constant-current charge to 1.5 V cell during the full-cell crossover experiment S11

Figure S6 : ^{1}H NMR spectrum of the catholyte from the middle of the 1.5 V voltage hold during the full-cell crossover experiment S11

Figure S7 : ^{1}H NMR spectrum of the catholyte from the end of the 1.5 V voltage hold during the full-cell crossover experiment S12

Figure S8 : ^{1}H NMR spectrum of the catholyte from the end of the 50 mA constant-current discharge during the full-cell crossover experiment S12

Figure S9 : ^{1}H NMR spectrum of the catholyte from the middle of the second cell rest period during the full-cell crossover experiment.. S13

Figure S10 : ^{1}H NMR spectrum of the catholyte from the end of the second cell rest period during the full-cell crossover experiment.. S13

Figure S11 : Photos of the orange-brown precipitates S14

Figure S12 : Calibrating the relationship between ferricyanide concentration and HOD chemical shift in ^{1}H NMR (9.4 T)... S15

Figure S13 : Photos of 2,6-DHAQ simulated-crossover solutions in NMR tubes before and after aging S16

Figure S14 :TOCSY spectrum of an aged solution of 2,6-DHAQ-spiked ferricyanide catholyte... S17

Figure S15 : DOSY spectrum of an aged solution of 2,6-DHAQ-spiked ferricyanide catholyte... S18

Figure S16 : Kinetic isotope effects on change in ferricyanide losses during 2,6-DHAQ on-line NMR simulated-crossover experiments.. S19

Figure S17 : Full dataset showing change in amount of ferricyanide during 2,6-DHAQ simulated-crossover experiment (in D_2O) and control experiment S19

Figure S18: Results from 2,6-DHAQ simulated-crossover experiments performed at different anolyte:catholyte mixing ratios S20

Figure S19 : DOSY and ^{1}H NMR spectra of an aged 2,6-DHAQ-spiked ferricyanide catholyte with an added formamide standard.. S21

Figure S20 : ^{1}H NMR spectra of a fresh ferrocyanide catholyte and 2,6-DHAQ anolyte S22

Figure S21 : ^{1}H NMR spectra of a ferricyanide catholyte before and after aging, highlighting the appearance of the hypothesised formamide peak......................... S23

Figure S22: Rate of formation of the formamide peak during the on-line 2,6-DHAQ simulated-crossover experiment.. S24

Figure S23 : Quantitative ^{1}H NMR spectra of 2,6-DHAQ simulated-crossover solution before and after aging S24

Figure S24 : Quantitative ^{1}H NMR spectra of 2,7-AQDS simulated-crossover solution before and after aging S25

Figure S25 : Quantitative ^{1}H NMR spectra of 2,6-DPPAQ simulated-crossover solution before and after aging. S25

Figure S26 : Quantitative ^{1}H NMR spectra of DHPS simulated-crossover solution before and after aging. S26

Figure S27 : Raman spectra of FeQ-solids and Fe-solids compared to standards S26

Figure S28 : PXRD of Fe-solids and FeQ-solids S27

Figure S29 : PXRD of FeQ-solids, prepared in 1.2 M KOH supporting electrolyte S27

Figure S30 : Hysteresis curves for the Fe-solids and FeQ-solids, measured by VSM S28

Figure S31 : ^{1}H NMR of the FeQ-solids dissolved in DCl S29

Figure S32 : Calibration curves for 2,6-DHAQ H_A S29

Tables...

Table S1 : Mean ^{1}H NMR chemical shift for the water peak (HOD) measured during *ex situ* simulated-crossover experiments S30

Table S2 : Mean percentage loss of ferricyanide measured indirectly from changes in HOD ^{1}H NMR chemical shift during *ex situ* simulated-crossover experiments S30

Table S3 : Mean ^{1}H NMR integral for each anthraquinone anolyte measured in ferricyanide electrolyte during *ex situ* simulated-crossover experiments S31

Table S4 : Mean percentage change in anthraquinone concentration measured during the *ex situ* simulated-crossover experiments S31

Table S5 : Summary table comparing each type of crossover experiment used in this study S32

Table S6 : Combustion and ICP-MS elemental analysis of Fe-solids, FeQ-solids and iron(III) oxide and iron (III) hydroxide standards.. S32

Schemes...

Scheme S1 : Proposed mechanism of ferricyanide loss and conversion to iron oxide hydroxide precipitates in the presence of 2,6-DHAQ^{2-}. S33

Experimental Methods S1: Quantitative on-line NMR acquisitions parameters

For the full-cell on-line NMR crossover experiments, pseudo-2D ^{1}H NMR experiments were performed by direct excitation with a 90° pulse on a 400 MHz standard bore Bruker magnet equipped with a 5 mm HD Smart Probe and Bruker Avance III spectrometer. All the redox-flow battery equipment was placed outside the 5 gauss (G) line. The 90° pulse length was 16.430 μs. Each spectrum was obtained by using 16 scans, a 1.917 s acquisition time, and 16.875 s recycle delay, resulting in a spectrum recorded every 5 min ((1.917 s + 16.875 s) × 16 = 300.7 s). The recycle delay chosen was quantitative for oxidised 2,6-DHAQ H_A throughout battery charging, and increasing the recycle delay did not change the integration of the signal. The quantitative recycle delay was selected based on our previous work, where the T_1 of 2,6-DHAQ H_A was measured in electrolytes prepared with different ferrocyanid:ferricyanide ratios, reflecting the composition changes expected within the catholyte during battery charging.[1] The T_1 of 2,6-DHAQ H_A was found to be less than 2.7 s under all conditions, therefore a recycle delay of 16.875 s was chosen, so that recycle delay > 5*T_1.[1]

Under flow, signal suppression can result due to T_1 relaxation effects, if the electrolyte does not reside in the magnet for long enough for equilibrium magnetisation to be developed. A longer T_1, means that a lower flow rate is needed to avoid signal suppression due to flow.[2] Here we are using a 90° pulse and quantitative recycle delay under all conditions, so provided the residence time of the sample in the magnet is greater than the quantitative recycle delay used in the experiment, we avoid signal suppression. Based on a sample volume within magnet of 0.5 mL, a flow rate of <2.2 mL min-1 should be used to avoid signal suppression of a proton resonance with a T_1 <2.7 s. Here we used a flow rate of 1.6 mL min^{-1}. We also experimentally validated that no signal suppression was observed under these conditions using variable flow rate experiments in our first publication describing the same on-line NMR methodology used here.[1]

For the on-line simulated-crossover experiments, the same on-line NMR acquisition method was used, except these were performed on a different spectrometer (400 MHz (9.4 T) wide-bore Bruker NMR spectrometer, equipped with a BBO 400 MHz W1S2 5mm Z-gradient diffusion probe). The 90° pulse length was 18.50-18.20 μs for the experiments performed in D_2O and 20.25 μs for the experiment performed in H_2O. Each spectrum was obtained by using 16 scans, a 1.917 s acquisition time, and 16.875 s recycle delay, resulting in a spectrum recorded every 5 min ((1.87 s + 16.90 s) × 16 = 300.32 s).

All spectra were referenced to the chemical shift 4.8 ppm using an external D_2O reference and the spectrometer lock was disabled. The probe temperature was set to 25 °C for all experiments. The room temperature was kept between 20-21 °C.

Experimental Methods S2: Calibration of 2,6-DHAQ concentration

The 2,6-DHAQ concentration was determined by integrating 2,6-DHAQ proton H_A. The signal was calibrated by making a calibration curve for a series 2,6-DHAQ solutions of known concentration (between 50 mM and 0.1 mM 2,6-DHAQ, see Figure S32). It is important to note that in these full-cell crossover experiments, we are quantifying the concentration of the oxidised form of 2,6-DHAQ only (2,6-$DHAQ^{2-}$). Based on previous work by Zhao *et al.*, we would expect the presence of the 2,6-$DHAQ^{3\bullet-}$ radical, even at low concentrations, to result in the complete loss of 2,6-$DHAQ^{2-}$ resonances H_A and H_B, due to rapid self-exchange electron-transfer reactions between 2,6-$DHAQ^{3\bullet-}$ and 2,6-$DHAQ^{2-}$.[3] In these experiments, we observe a gradual loss of all 2,6-$DHAQ^{2-}$ ^1H resonances at the same rate, indicating that 2,6-$DHAQ^{3\bullet-}$ is not present in the catholyte that is being analysed by ^1H NMR. This observation can be understood by considering the conditions in which the 2,6-DHAQ is subjected to in the battery environment. Though 2,6-$DHAQ^{3\bullet-}$ and the fully reduced form of 2,6-DHAQ (2,6-$DHAQ^{4-}$) are being formed in the anolyte side during battery charging, when these reduced forms of 2,6-DHAQ pass through the membrane into the catholyte side, they are oxidised by the excess of ferricyanide present, reforming 2,6-$DHAQ^{2-}$.

Similarly, for the *ex situ* and on-line simulated-crossover experiments we are quantifying the concentration of 2,6-$DHAQ^{2-}$ only. For the simulated-crossover experiments, 2,6-$DHAQ^{3\bullet-}$ would not be expected to form, as the anolyte was prepared using the oxidised form of 2,6-DHAQ (2,6-$DHAQ^{2-}$), and there are no species present in the anolyte or catholyte that would be expected to reduce 2,6-$DHAQ^{2-}$ to form the 2,6-$DHAQ^{3\bullet-}$ radical or 2,6-$DHAQ^{4-}$. Similarly, the chemical shift separation between 2,6-DHAQ proton H_A and H_C (~1.1 ppm) remains constant, further confirming that we are only detecting 2,6-$DHAQ^{2-}$ on the catholyte side, consistent with previous work.[1,3] In contrast, the peak separation between the protons in the same position in 2,6-$DHAQ^{4-}$ is ~1.5 ppm.[3]

Experimental Methods S3: Calibration of ferricyanide concentration

The ferricyanide concentrations were measured indirectly from the change in water ^1H NMR chemical shift, as described in our previous work,[1] inspired by the work by Zhao *et al.*[4] The relationship between concentration of paramagnetic species and change in HOD chemical

shift depends on the effective magnetic moment. For ferricyanide, the effective magnetic moment is higher than the spin-only value, due to orbital contributions.[3] The relationship between ferricyanide concentration and HOD chemical shift was therefore experimentally determined using a series of catholyte solutions with different ferrocyanide:ferricaynide ratios (1:0, 2:1, 1:2 and 0:1, as shown in Figure S12).[1,4,5] The concentration of ferricyanide was then calculated assuming it is the only paramagnetic species in solution, and that the effective magnetic moment did not change.

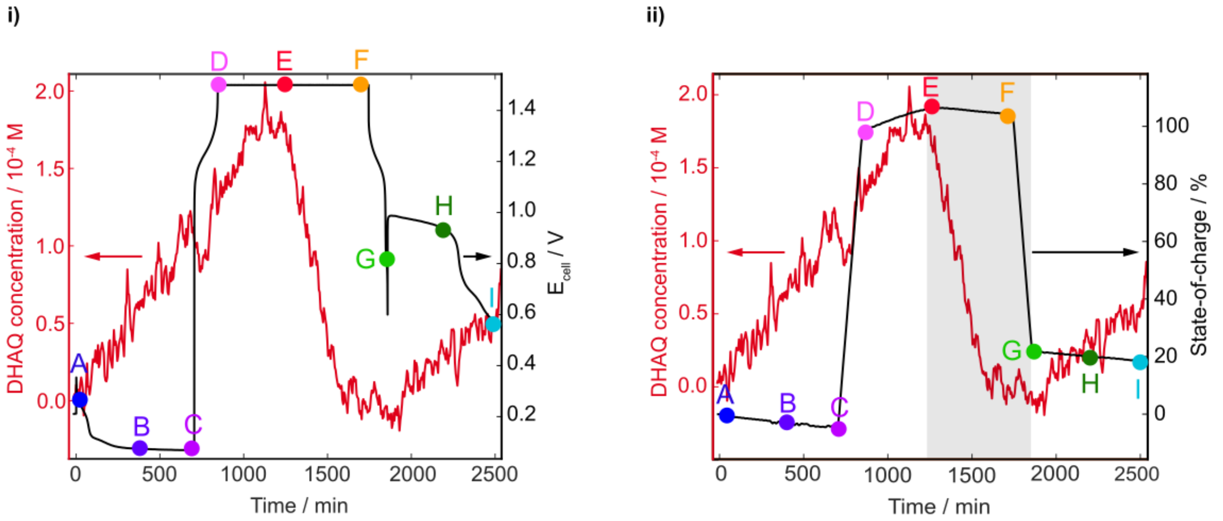

Figure S1: Full-cell simulated-crossover data (Experimental Methods 2), with coloured spots and letters (A-I) indicating the timepoints used for the ^{1}H NMR spectra shown in Figures S2-S10. The 2,6-DHAQ crossover data (red trace) is plotted along with i) the cell voltage (E_{cell}), and ii) the battery state-of-charge (which is determined from the measured ferricyanide concentration).

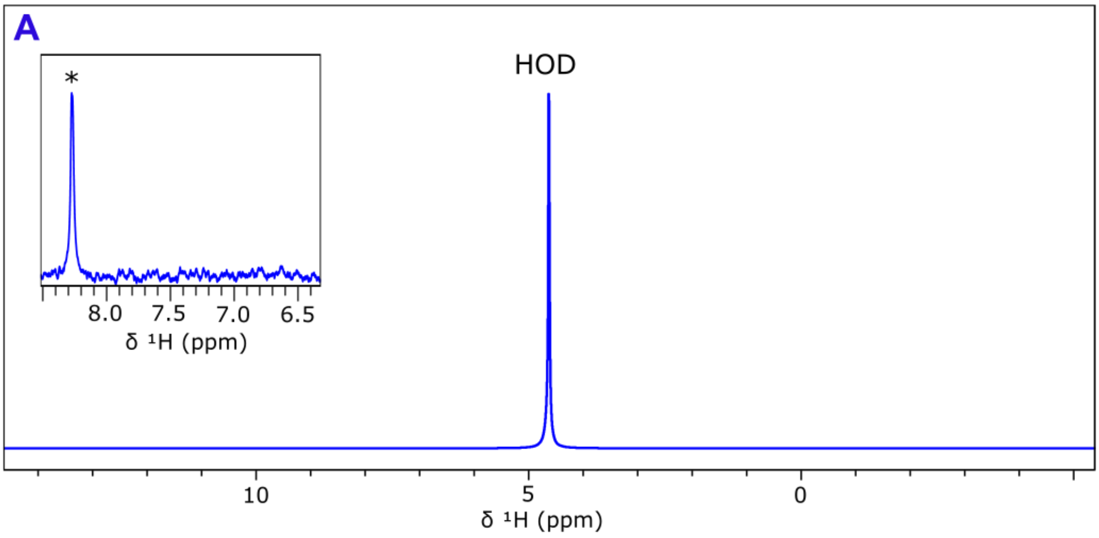

Figure S2: ^{1}H NMR spectrum of the catholyte from the start of the full-cell crossover experiment, corresponding to Figure S1, slice A. The inset shows the ^{1}H NMR spectrum expanded in the region of interest. The formamide peak is indicated with an asterisk.

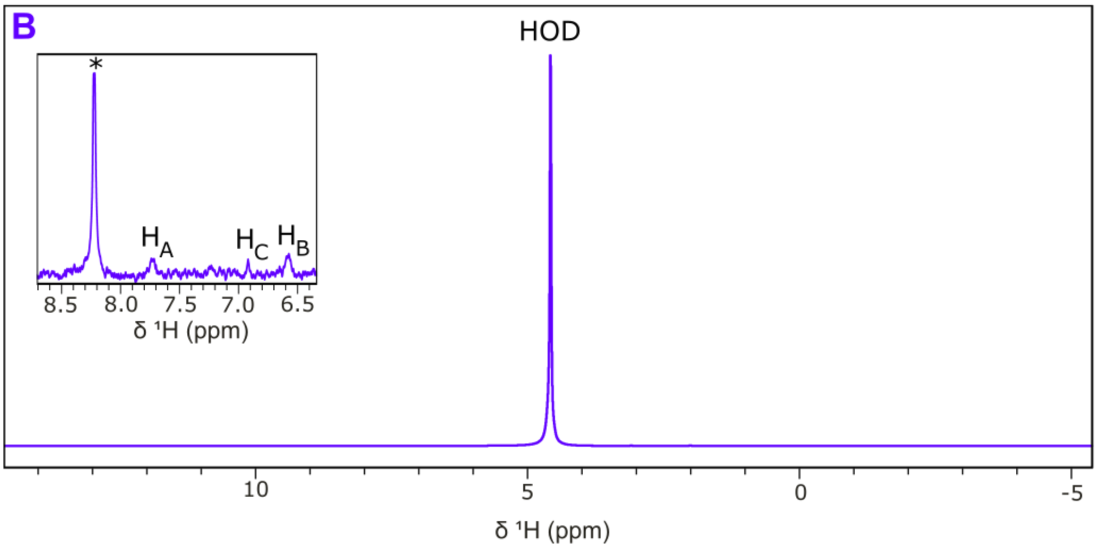

Figure S3: ¹H NMR spectrum of the catholyte from the middle of the first cell rest period during the full-cell crossover experiment, corresponding to Figure S1, slice B. The inset shows the ¹H NMR spectrum expanded in the region of interest. The 2,6-DHAQ ¹H NMR resonance peaks are assigned as H_A, H_B and H_C. The formamide peak is indicated with an asterisk.

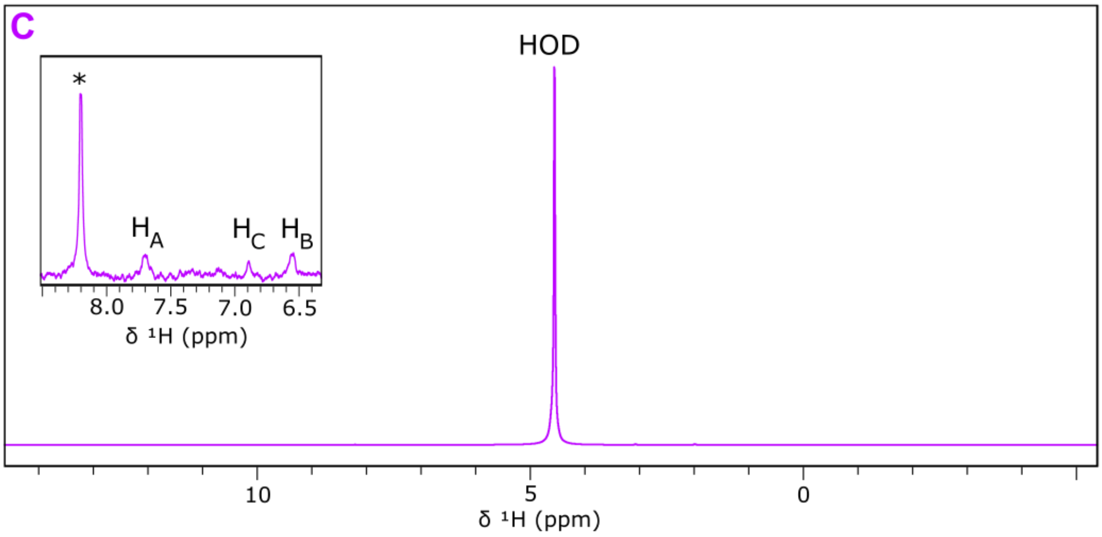

Figure S4: ¹H NMR spectrum of the catholyte from the end of the first cell rest period during the full-cell crossover experiment, corresponding to Figure S1, slice C. The inset shows the ¹H NMR spectrum expanded in the region of interest. The 2,6-DHAQ ¹H NMR resonance peaks are assigned as H_A, H_B and H_C. The formamide peak is indicated with an asterisk.

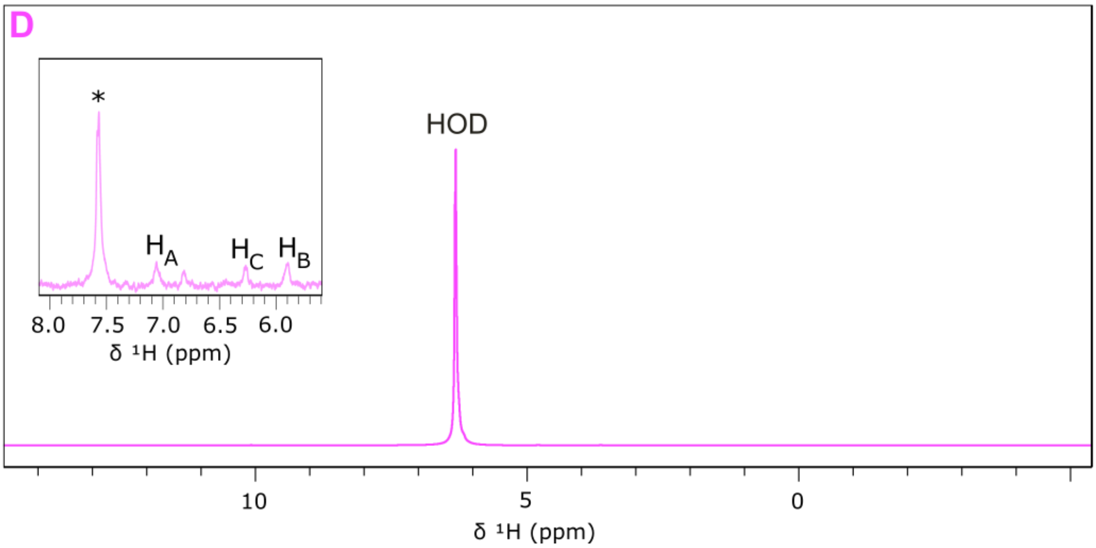

Figure S5: ^{1}H NMR spectrum of the catholyte from the end of the 50 mA constant-current charge to 1.5 V cell during the full-cell crossover experiment, corresponding to Figure S1, slice D. The inset shows the ^{1}H NMR spectrum expanded in the region of interest. The 2,6-DHAQ ^{1}H NMR resonance peaks are assigned as H_A, H_B and H_C. The formamide peak is indicated with an asterisk.

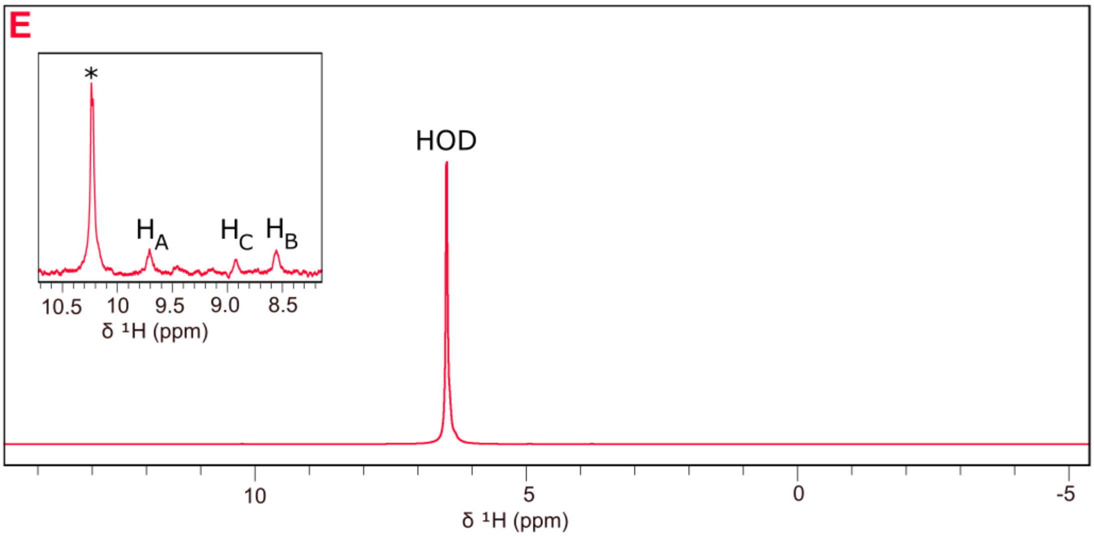

Figure S6: ^{1}H NMR spectrum of the catholyte from the middle of the 1.5 V voltage hold during the full-cell crossover experiment, corresponding to Figure S1, slice E. The inset shows the ^{1}H NMR spectrum expanded in the region of interest. The 2,6-DHAQ ^{1}H NMR resonance peaks are assigned as H_A, H_B and H_C. The formamide peak is indicated with an asterisk.

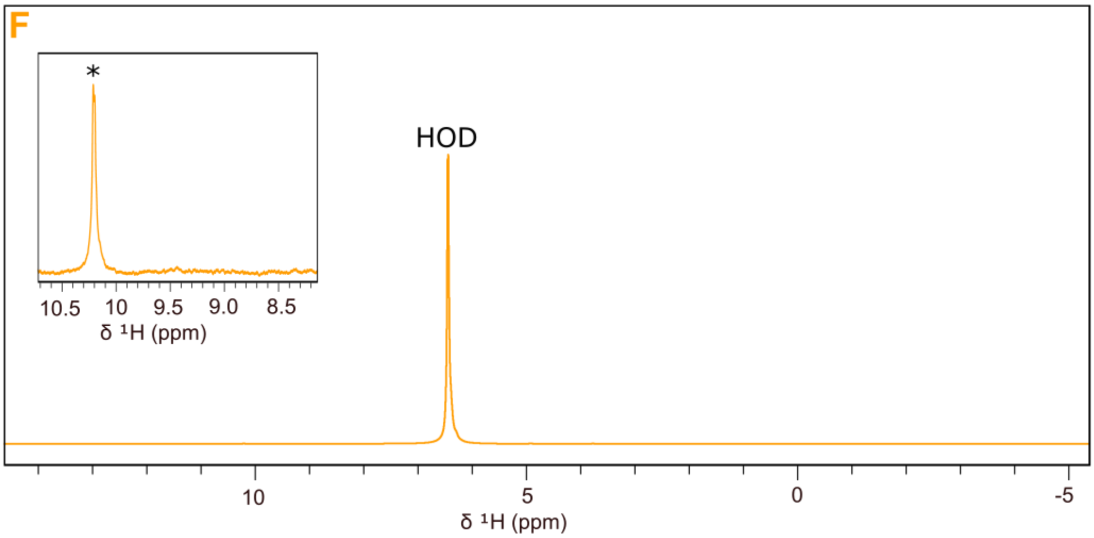

Figure S7: ^{1}H NMR spectrum of the catholyte from the end of the 1.5 V voltage hold during the full-cell crossover experiment, corresponding to Figure S1, slice F. The inset shows the ^{1}H NMR spectrum expanded in the region of interest. The formamide peak is indicated with an asterisk.

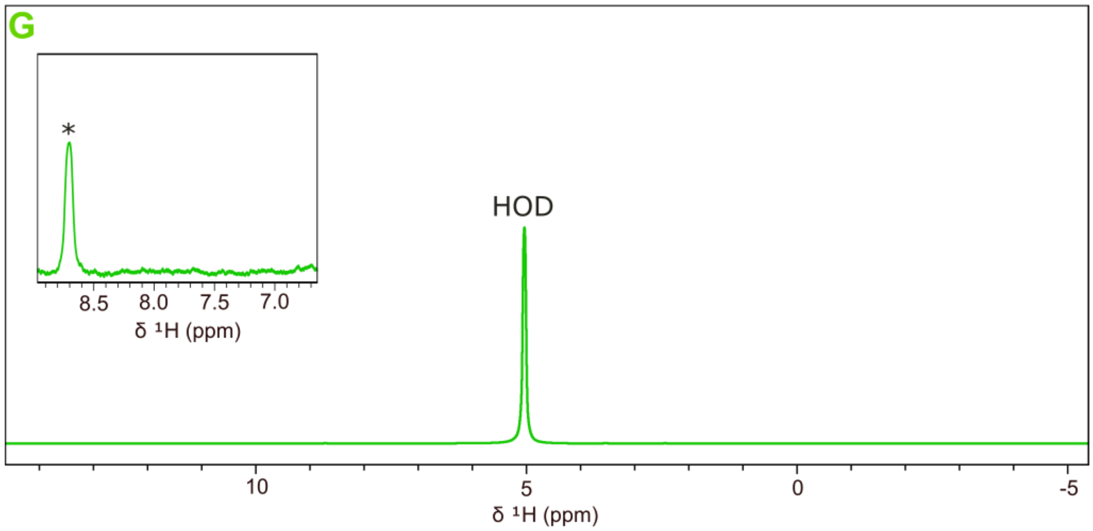

Figure S8: ^{1}H NMR spectrum of the catholyte from the end of the 50 mA constant-current discharge during the full-cell crossover experiment, corresponding to Figure S1, slice G. The inset shows the ^{1}H NMR spectrum expanded in the region of interest. The formamide peak is indicated with an asterisk.

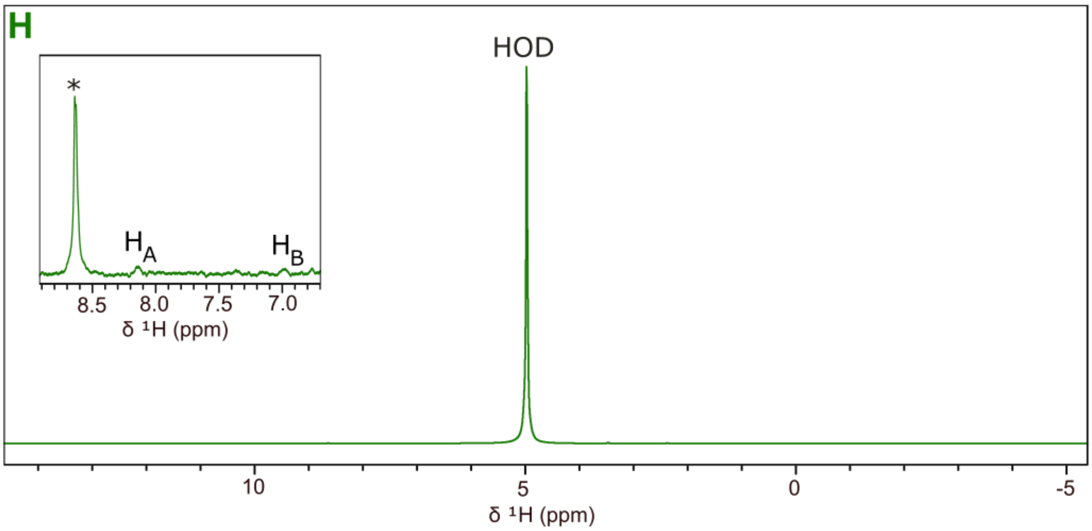

Figure S9: ^{1}H NMR spectrum of the catholyte from the middle of the second cell rest period during the full-cell crossover experiment, corresponding to Figure S1, slice H. The inset shows the ^{1}H NMR spectrum expanded in the region of interest. The 2,6-DHAQ ^{1}H NMR resonance peaks are assigned as H_A and H_B. Note that proton H_C is no longer visible, which is a result of H-D exchange when in the charged/reduced form of 2,6-DHAQ (2,6-DHAQ^{4-}). Although 2,6-DHAQ is oxidised back to the 2,6-DHAQ^{2-} form on crossing through to the catholyte side, the H_C protons have been replaced with deuterium and are no longer detectable by ^{1}H NMR.[1,3] The formamide peak is indicated with an asterisk.

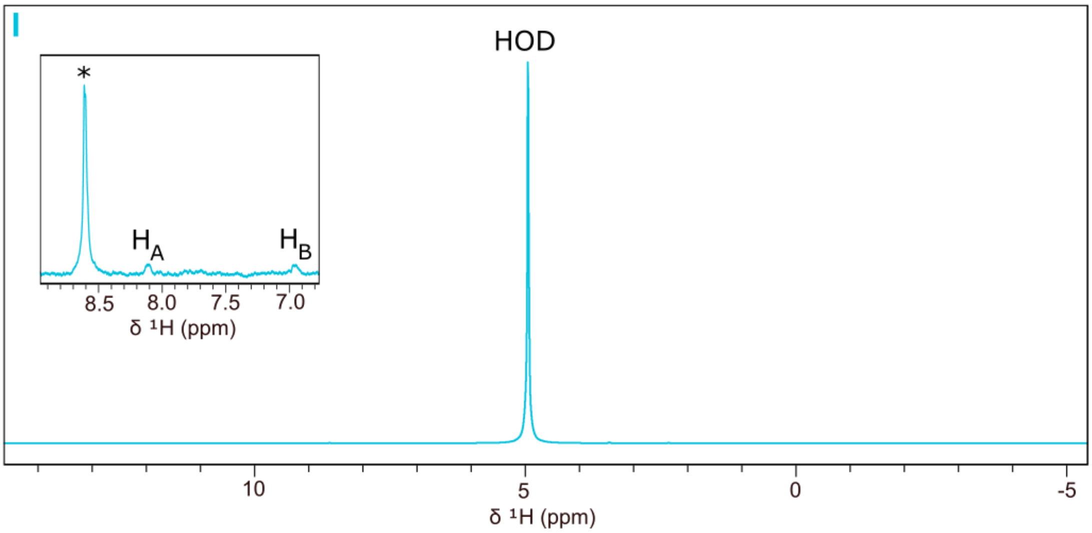

Figure S10: ^{1}H NMR spectrum of the catholyte from the end of the second cell rest period during the full-cell crossover experiment, corresponding to Figure S1, slice I. The inset shows the ^{1}H NMR spectrum expanded in the region of interest. The 2,6-DHAQ ^{1}H NMR resonance peaks are assigned as H_A and H_B. The formamide peak is indicated with an asterisk.

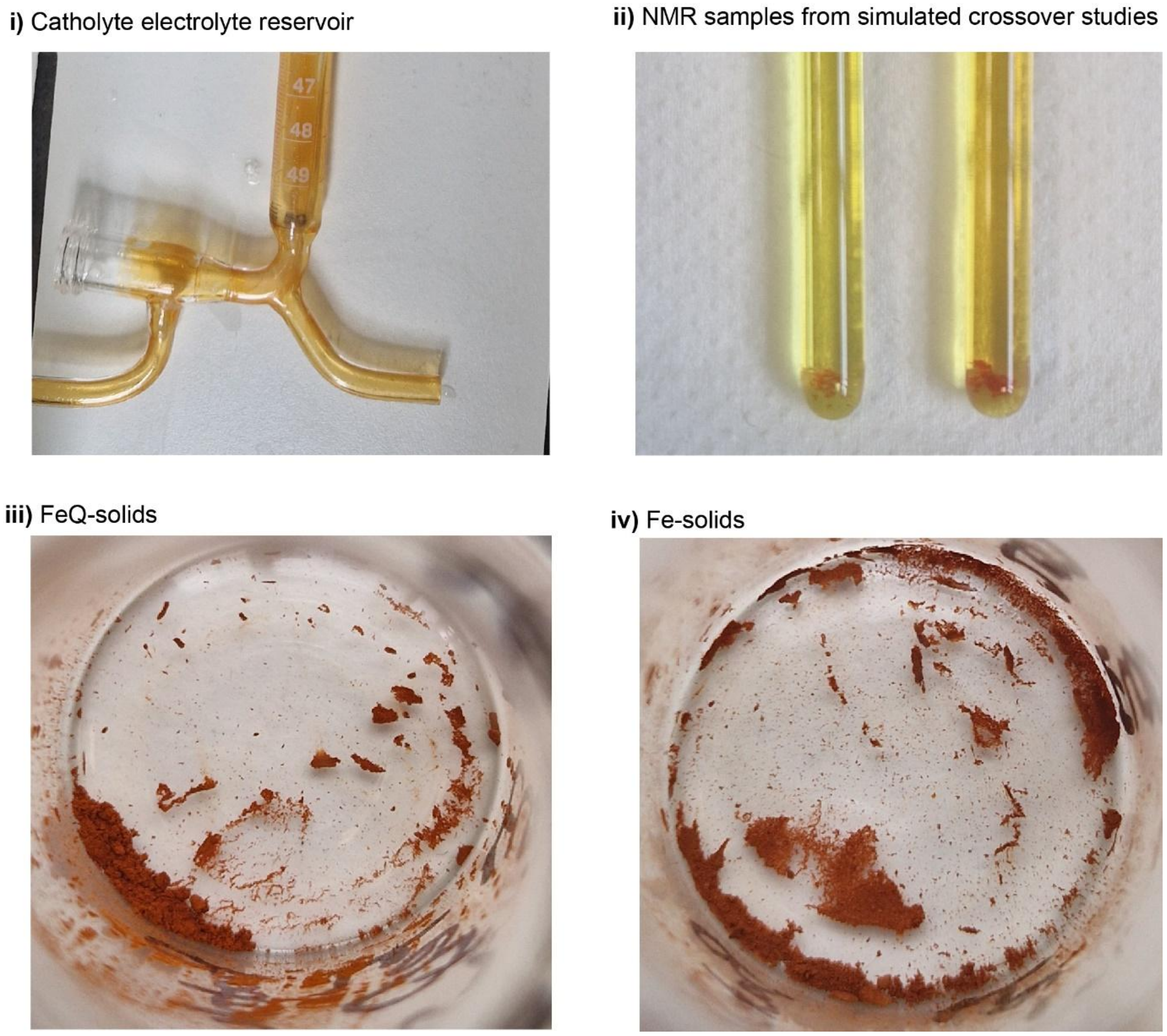

Figure S11: Photos of orange-brown precipitates. i) precipitates formed in the 2,6-DHAQ/ferrocyanide battery, coating the redox-flow battery catholyte reservoir during the full-cell experiment (Experimental Methods 1 and 2). ii) solid precipitates found at the bottom of NMR tubes from the simulated-crossover experiments, where aged ferricyanide is shown on the left and aged 2,6-DHAQ-spiked ferricyanide is shown on the right (Experimental Methods 3). iii) solid precipitates formed from 2,6-DHAQ spiked ferricyanide catholyte prepared in 1 M KOH/H_2O (FeQ-solids, prepared as described in Experimental Methods 8). iv) solid precipitates formed from ferricyanide catholyte prepared in 1 M KOH/H_2O (Fe-solids, prepared as described in Experimental Methods 8).

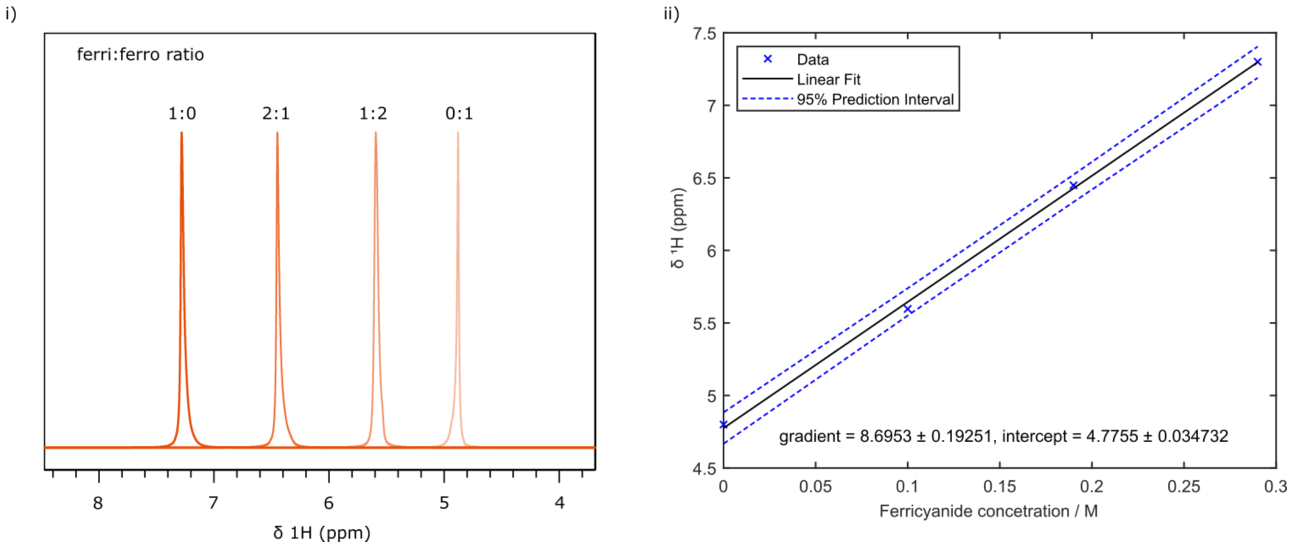

Figure S12. Calibrating the relationship between ferricyanide concentration and HOD chemical shift in ^{1}H NMR (9.4 T). i) ^{1}H NMR spectra of catholytes with varying ferricyanide:ferrocyanide ratios, where the total concentration of iron-containing species remains consonant at 0.29 M. Note that the intensity of the spectra have been scaled to set the peak of maximum intensity to the same height. ii) Plot showing the relationship between HOD chemical shift and ferricyanide concentration. Panel ii) is adapted from a figure by Latchem *et al.*[1]

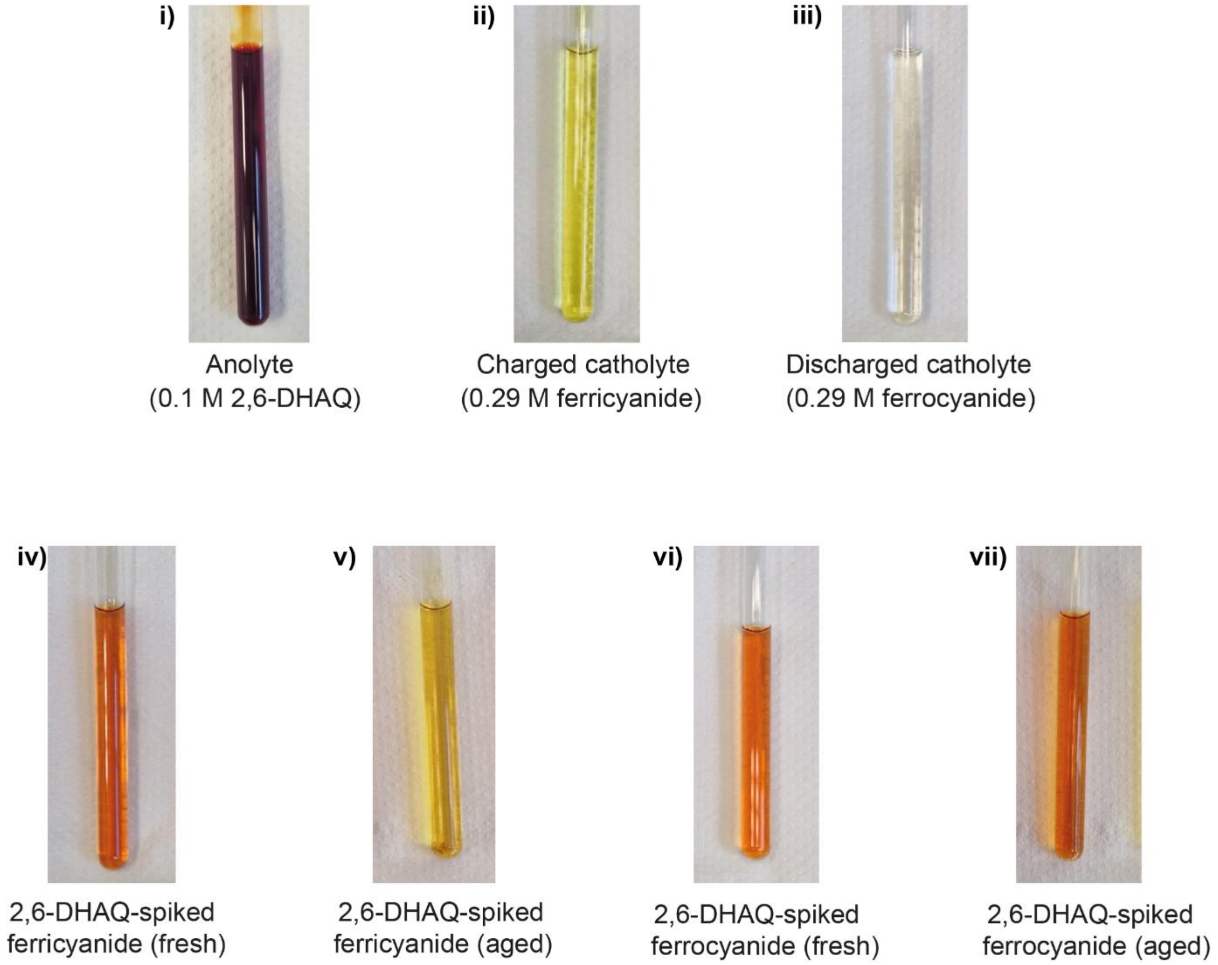

Figure S13: Photos of 2,6-DHAQ simulated-crossover solutions in NMR tubes before and after aging (from simulated-crossover experiments, as described in Experimental Methods 3). Photos show i) 0.1 M 2,6-DHAQ anolyte and ii) 0.29 M ferricyanide catholyte (fresh), iii) 0.29 M ferrocyanide catholyte (fresh), iv) ferricyanide catholyte spiked with ~2 mM 2,6-DHAQ (fresh), v) ferricyanide catholyte spiked with ~2 mM 2,6-DHAQ (1 week aged), vi) ferrocyanide catholyte spiked with ~2 mM 2,6-DHAQ (fresh), vii) ferrocyanide catholyte spiked with ~2 mM 2,6-DHAQ (1 week aged).

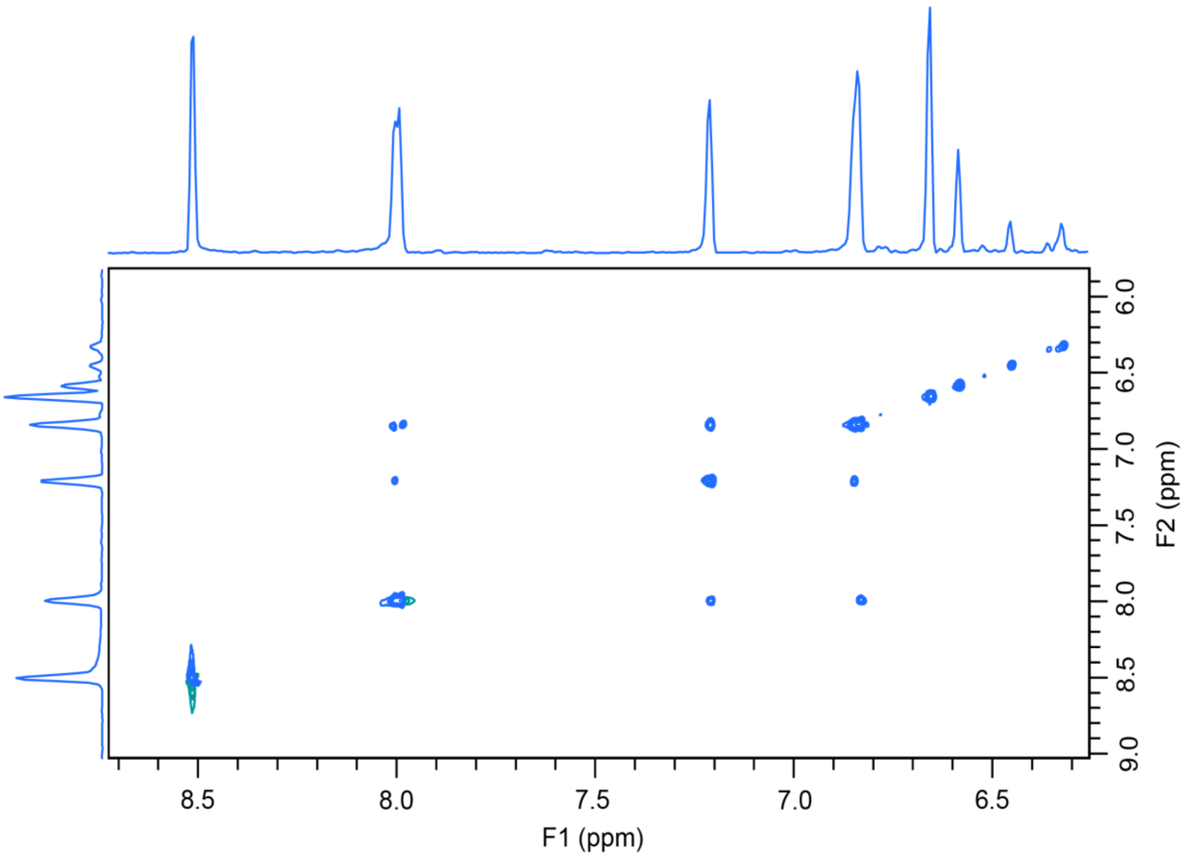

Figure S14: TOCSY spectrum of an aged 2,6-DHAQ-spiked ferricyanide catholyte. The spectrum was collected at 14.1 T. Note that the spectrometer lock was used during these measurements, so the chemical shift of the 2,6-DHAQ resonances and new peaks in this spectrum do not match those shown in Figure 3b, where the spectrometer lock was switched off. Here, H_A, H_B and H_C appear at 8 ppm, 6.8 ppm and 7.2 ppm, respectively.

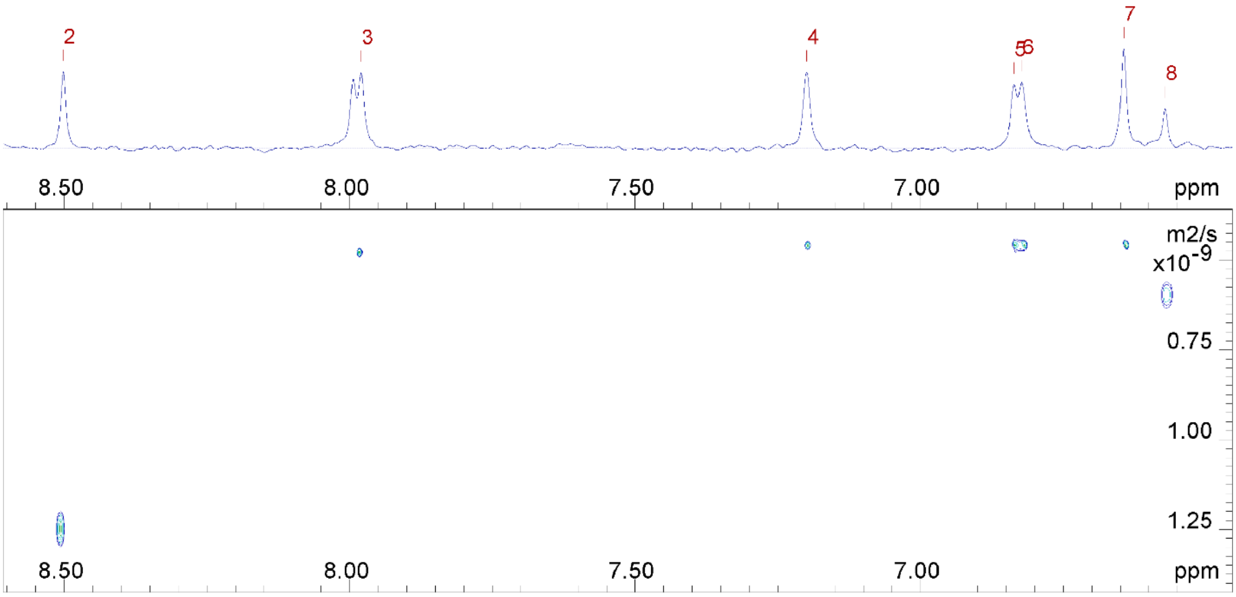

Figure S15: DOSY spectrum of an aged 2,6-DHAQ-spiked ferricyanide catholyte**.** The spectrum was collected at 14.1 T. Note that the spectrometer lock was used during these measurements, so the chemical shift of the 2,6-DHAQ resonances and new peaks in this spectrum do not match those shown in Figure 3b, where the spectrometer lock was switched off. Here, H_A, H_B and H_C appear at 8 ppm, 6.8 ppm and 7.2 ppm, respectively.

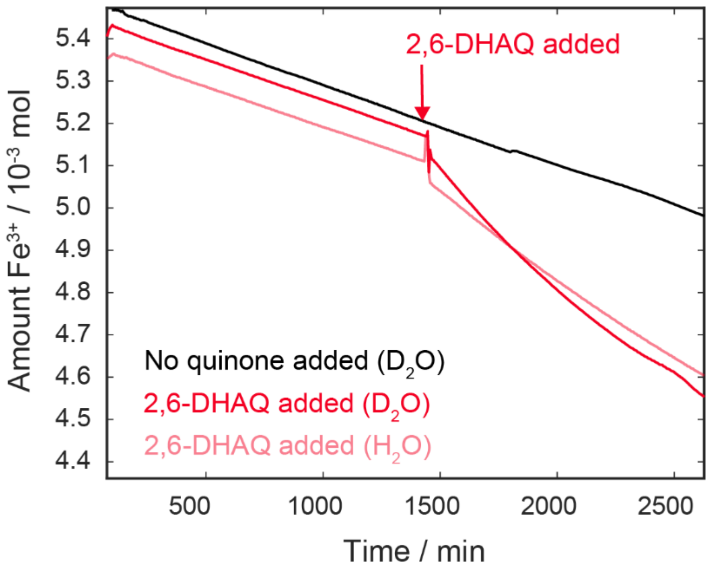

Figure S16: Kinetic isotope effects on ferricyanide losses during 2,6-DHAQ simulated-crossover experiments. Experiments were conducted twice, using either D_2O (dark red trace) or H_2O (light red trace) as the solvent.

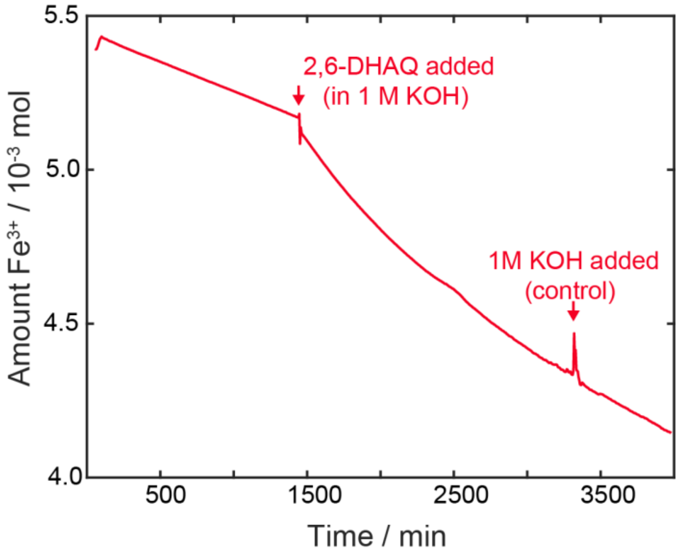

Figure S17: Full dataset showing change in amount of ferricyanide during the on-line 2,6-DHAQ simulated-crossover experiment and control experiment (in D_2O). The control was conducted by adding 0.4 mL of 1 M KOH (Experimental Methods 4), as indicated by the arrow on the graph.

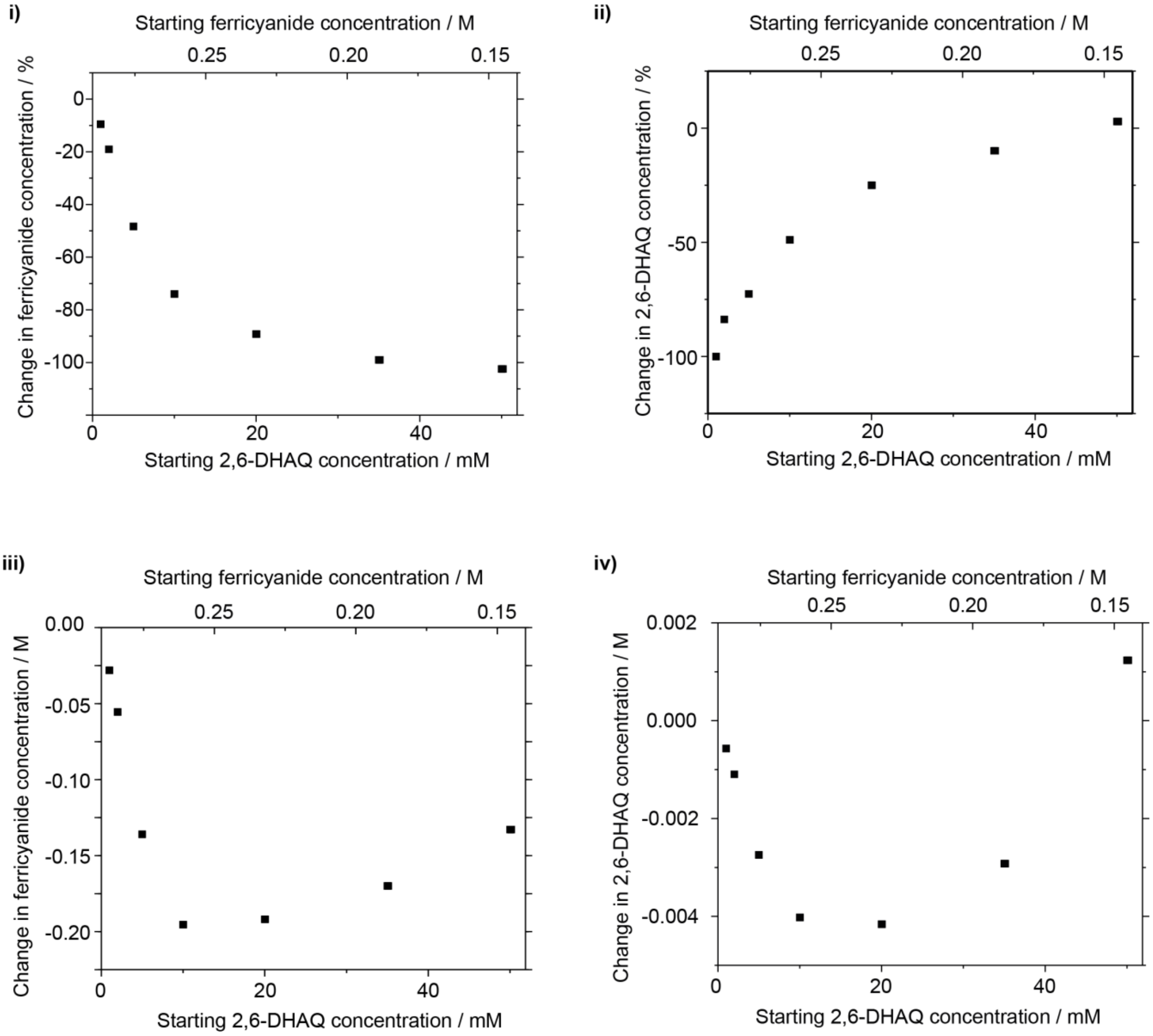

Figure S18: Results from concentration-varied 2,6-DHAQ simulated-crossover experiments. The percentage and molar losses are shown as a function of 2,6-DHAQ and ferricyanide starting concentration. Plots i) and ii) show the percentage losses for ferricyanide and 2,6-DHAQ, respectively. Plots iii) and iv) show the concentration drops for ferricyanide and 2,6-DHAQ, respectively.

i)

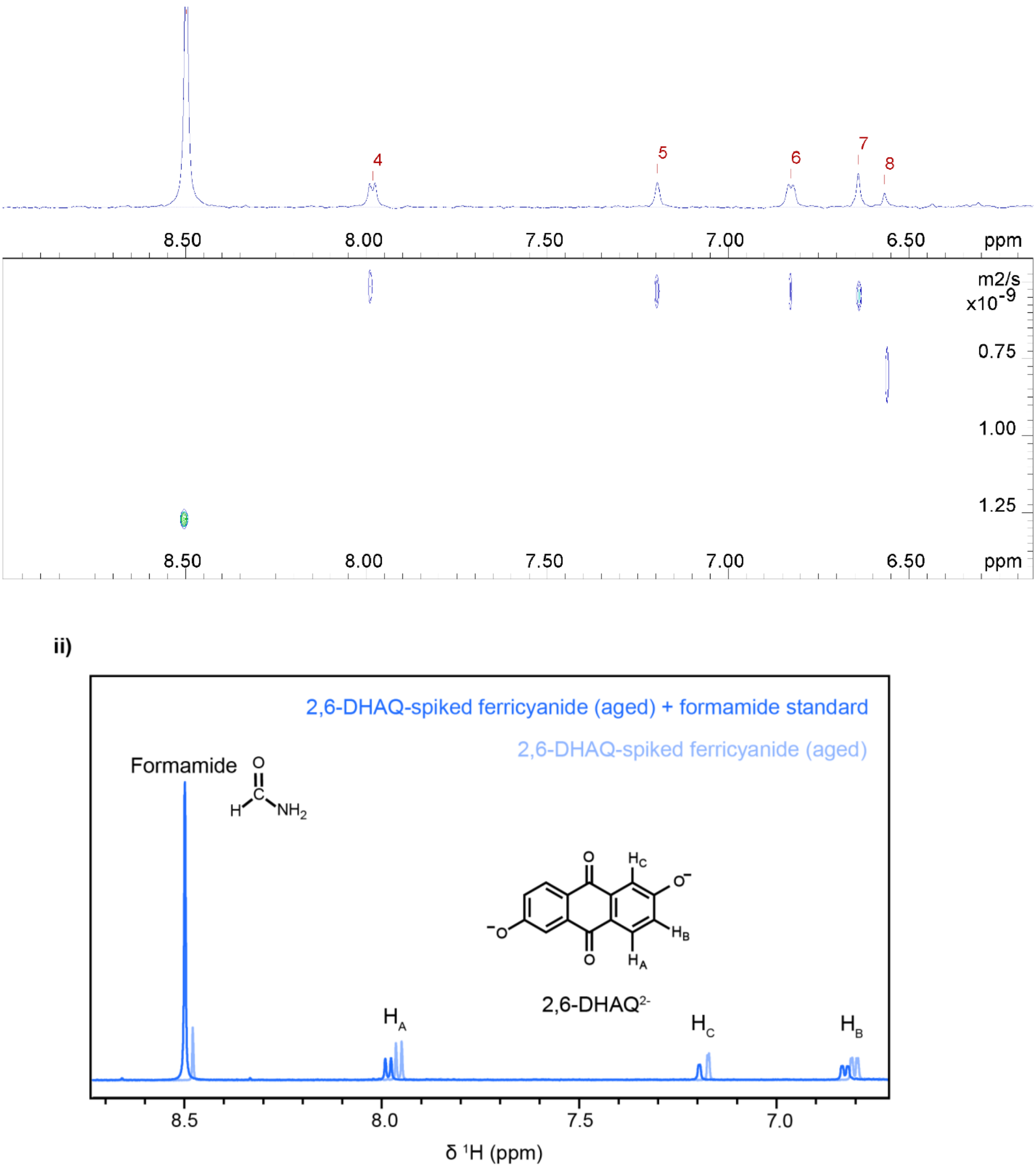

Figure S19: NMR spectra of an aged ferricyanide catholyte spiked with ~2 mM 2,6-DHAQ and a formamide standard. Spectra show i) DOSY of an aged ferricyanide catholyte spiked with ~2 mM 2,6-DHAQ with a formamide standard (see Figure S4 for DOSY spectra of the sample solution before addition of formamide) and ii) ^{1}H NMR spectra of the solution before and after addition of a formamide standard. All spectra were collected at 14.1 T.

i)

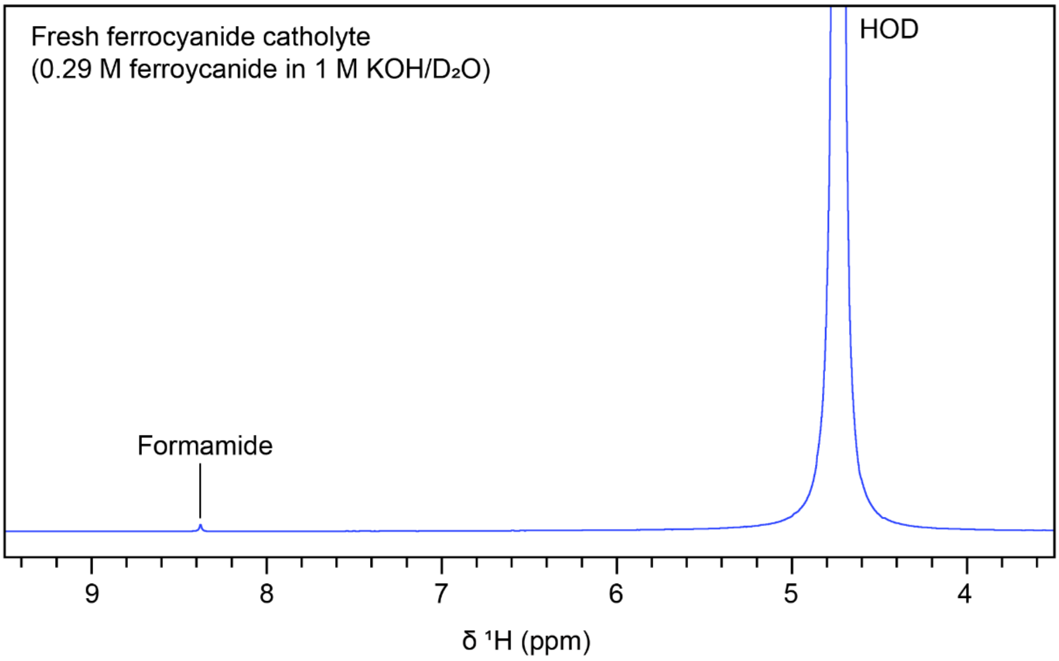

ii)

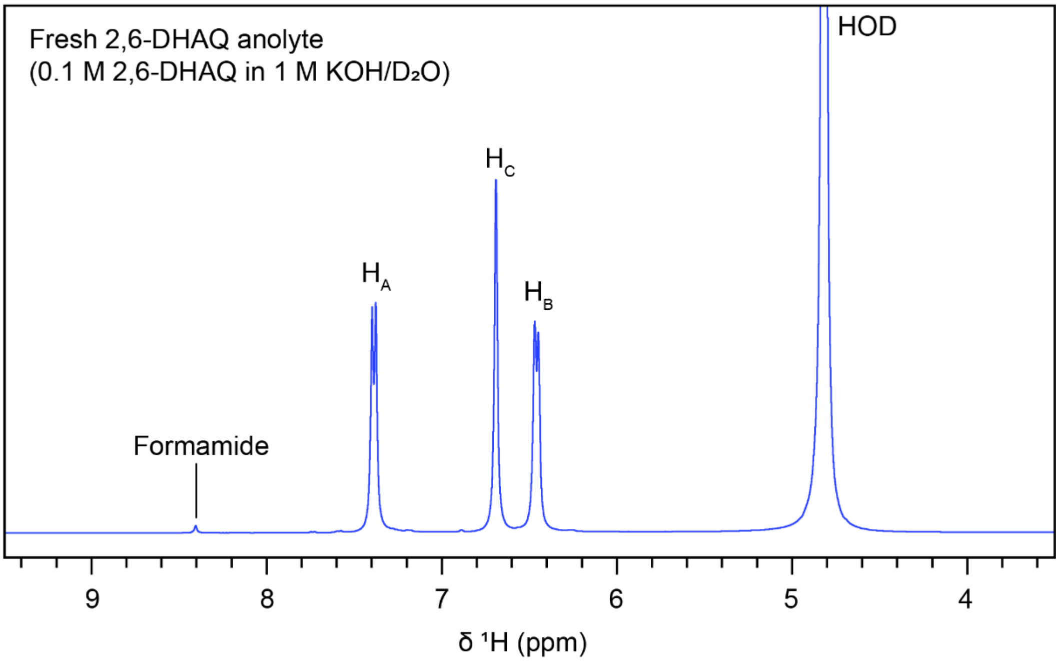

Figure S20: ^{1}H NMR spectra of fresh catholyte and anolytes, highlighting the peak that has been assigned to formamide. i) ^{1}H NMR spectrum of 0.29 M ferricyanide in 1 M KOH, ii) ^{1}H NMR spectrum of 0.1 M 2,6-DHAQ in 1 M KOH. All spectra were collected at 9.4 T.

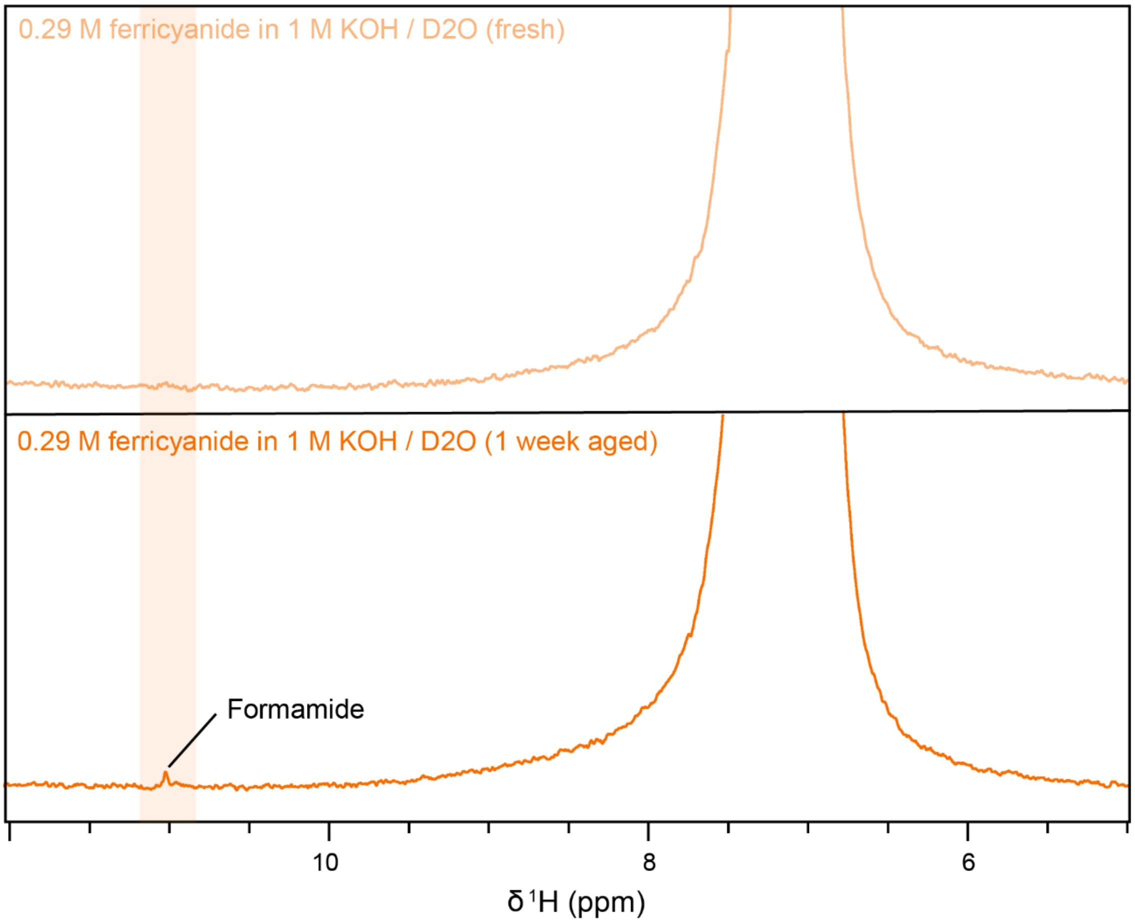

Figure S21: ^{1}H NMR spectra of a ferricyanide catholyte, highlighting the formation of a new peak that has been assigned to formamide. The formamide peak is not seen in the ^{1}H NMR spectra of the fresh ferricyanide catholyte, but it is seen after aging. All spectra were collected at 9.4 T.

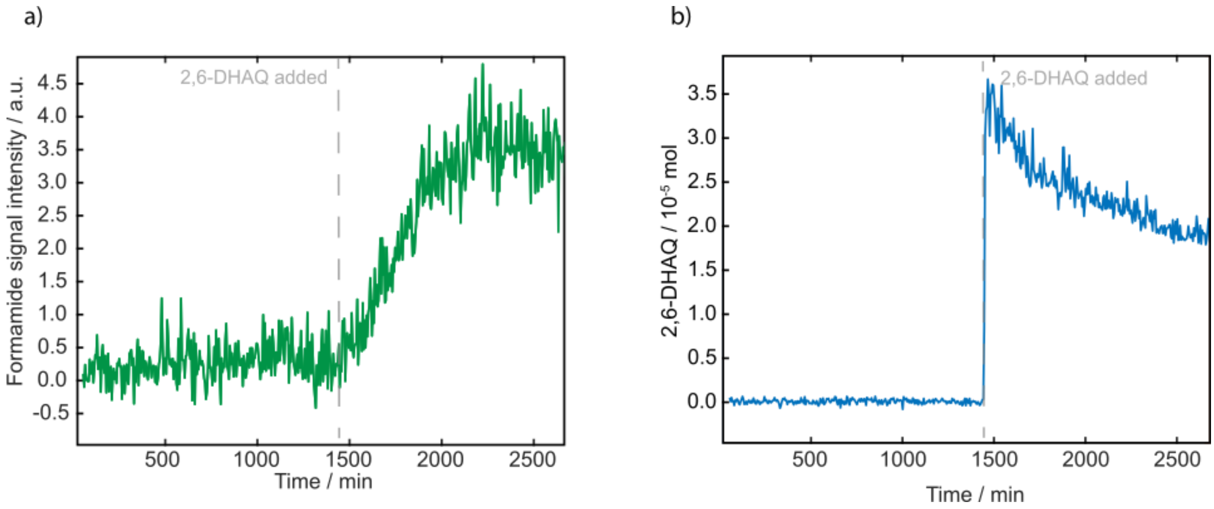

Figure S22: Rate of appearance of a) formamide and b) 2,6-DHAQ in the on-line ^{1}H NMR 2,6-DHAQ simulated-crossover experiment. The grey dashed line indicates the point when 2,6-DHAQ was added to the catholyte.

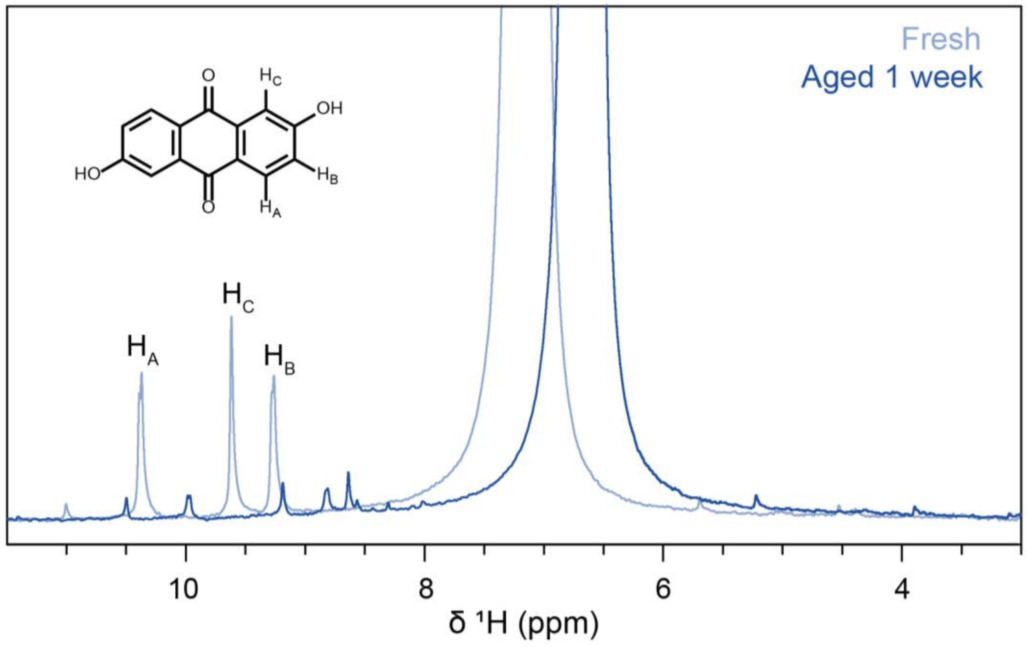

Figure S23: Quantitative ^{1}H NMR spectra of 0.29 M ferricyanide spiked with ~2 mM 2,6-DHAQ, all prepared in 1 M KOH/D_2O. The ^{1}H NMR of the fresh solution is shown in pale blue and aged solution in dark blue. All spectra were collected at 9.4 T.

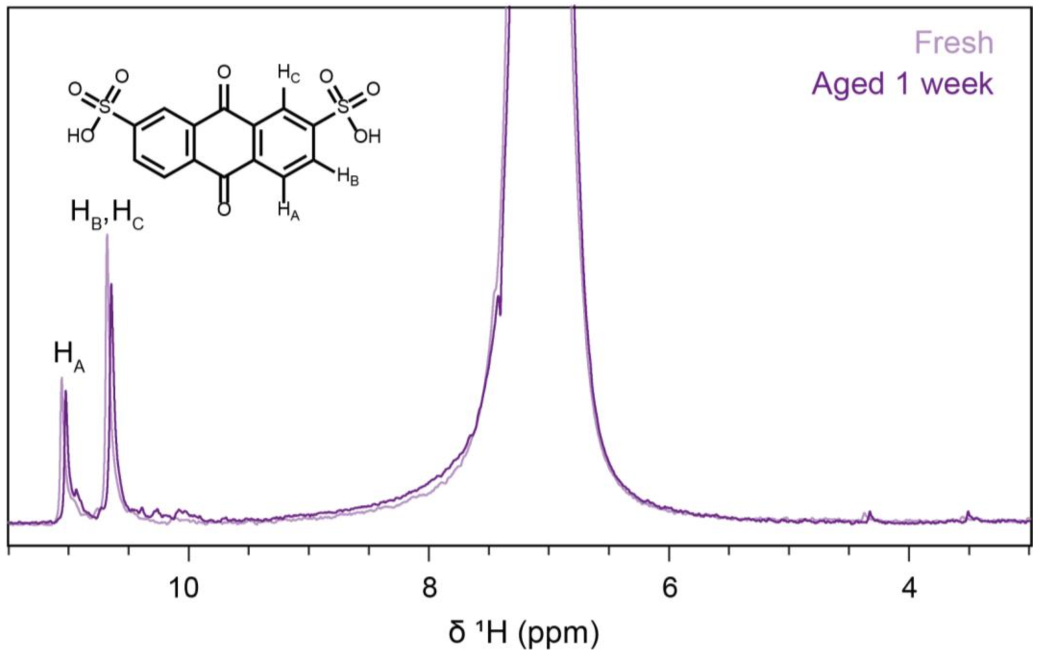

Figure S24: Quantitative ^1H NMR spectra of 0.29 M ferricyanide spiked with ~2 mM 2,7-AQDS, all prepared in 1 M KOH/D_2O. The ^1H NMR of the fresh solution is shown in pale purple and aged solution in dark purple. All spectra were collected at 9.4 T.

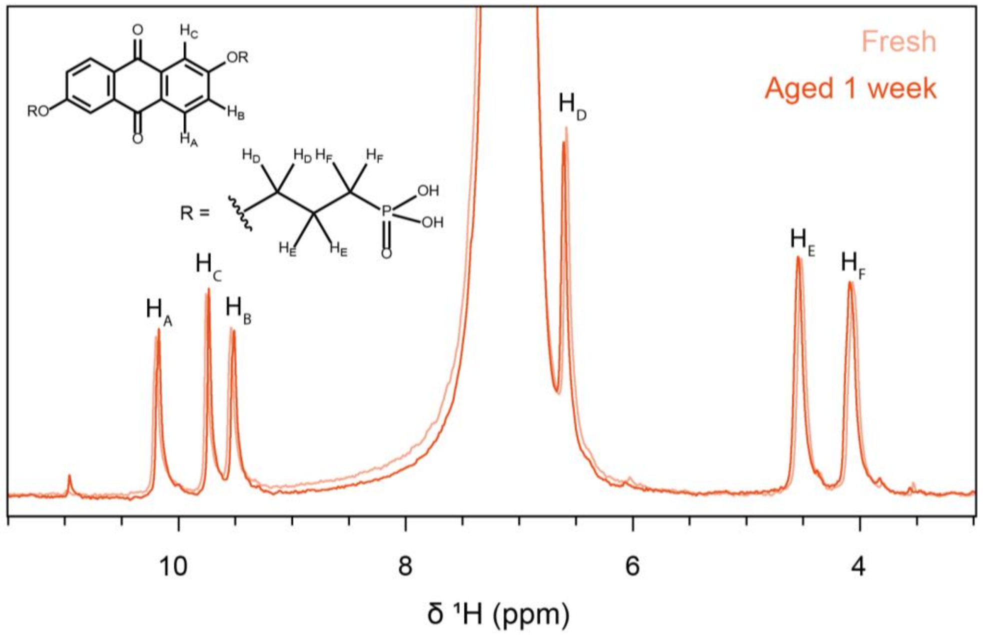

Figure S25: Quantitative ^1H NMR spectra of 0.29 M ferricyanide spiked with ~2 mM 2,6-DPPAQ, all prepared in 1 M KOH/D_2O. The ^1H NMR of the fresh solution is shown in pale orange and aged solution in dark orange. All spectra were collected at 9.4 T.

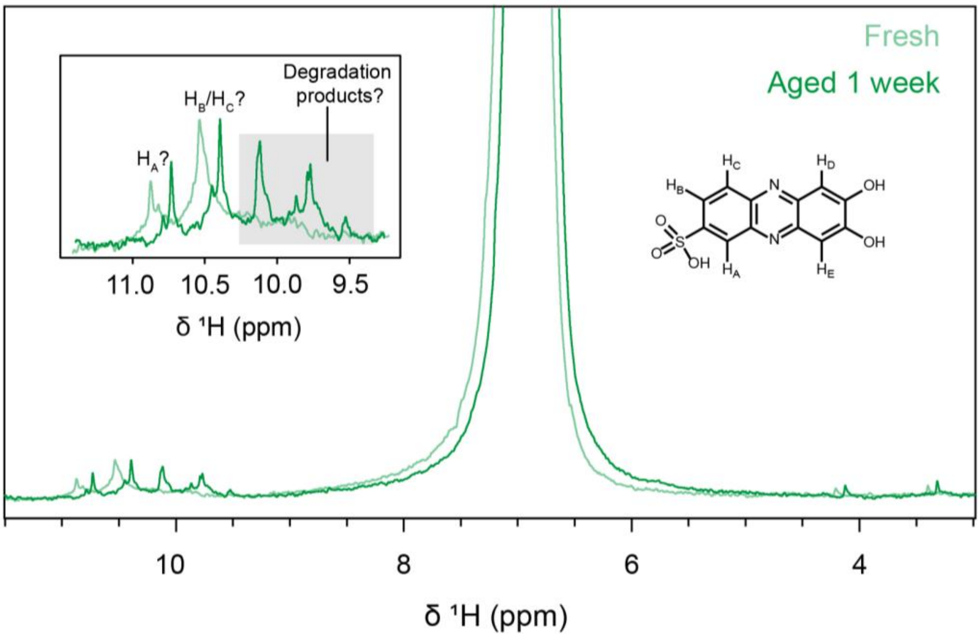

Figure S26: Quantitative ^{1}H NMR spectra of 0.29 M ferricyanide spiked with ~2 mM DHPS, all prepared in 1 M KOH/D_2O. The ^{1}H NMR of the fresh solution is shown in pale green and aged solution in dark green. All spectra were collected at 9.4 T.

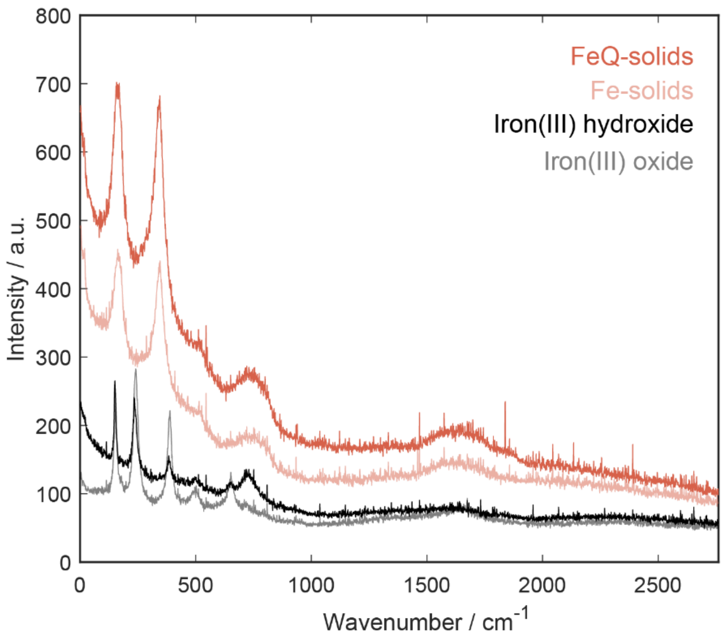

Figure S27: Raman spectra of the orange-brown precipitates. The Raman spectra for the FeQ-solids and Fe-solids have been plotted together with the Raman spectra for iron(III) oxide and iron(III) hydroxide standards.

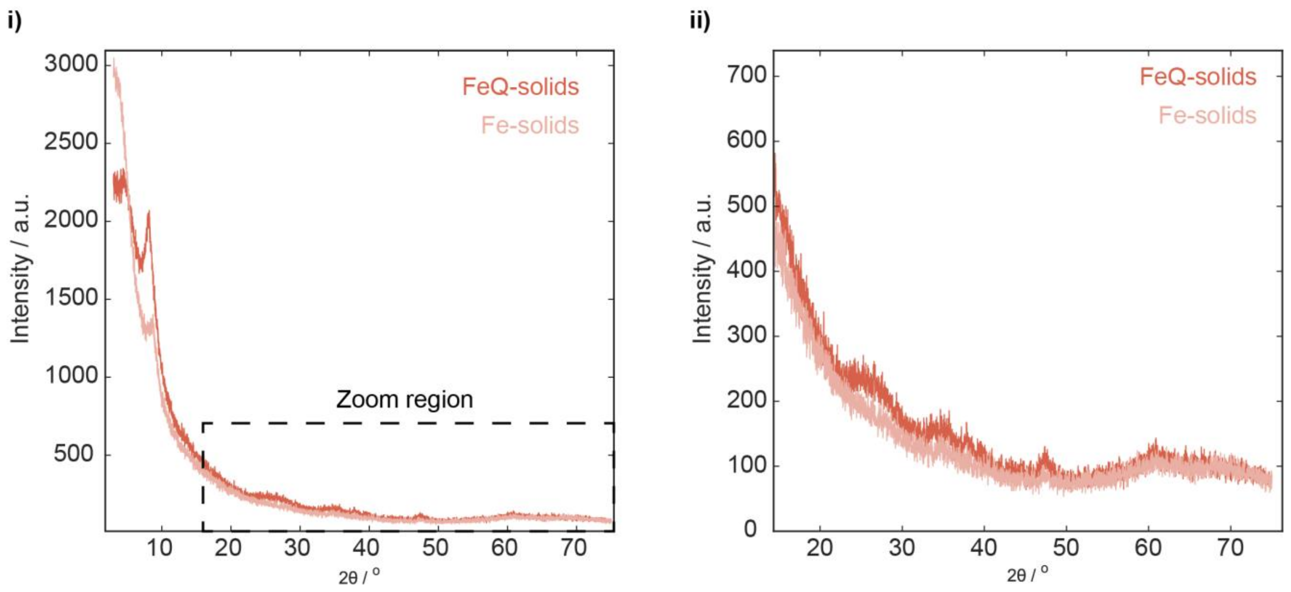

Figure S28: PXRD of FeQ-solids and Fe-solids, both prepared using 1 M KOH supporting electrolyte. i) full PXRD patterns for both the Fe-solids and FeQ-solids. ii) the PXRD patterns for both the Fe-solids and FeQ-solids, enlarged in the 16-25° region (as indicated by the black dashed box in i)).

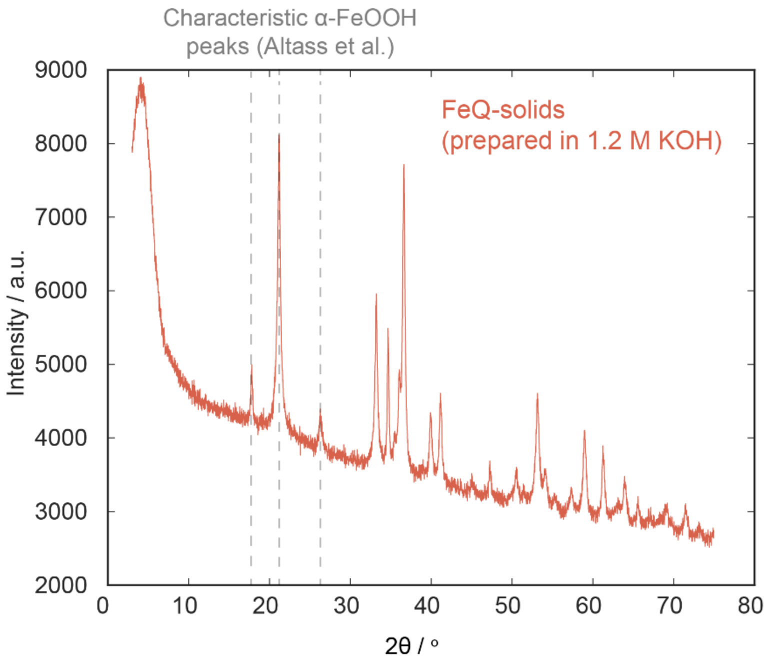

Figure S29: PXRD pattern of FeQ-solids formed in 1.2 M KOH supporting electrolyte. The key peaks from reference PXRD pattern of α-FeOOH (Altass et al.[6]) are indicated with grey dashed lines.

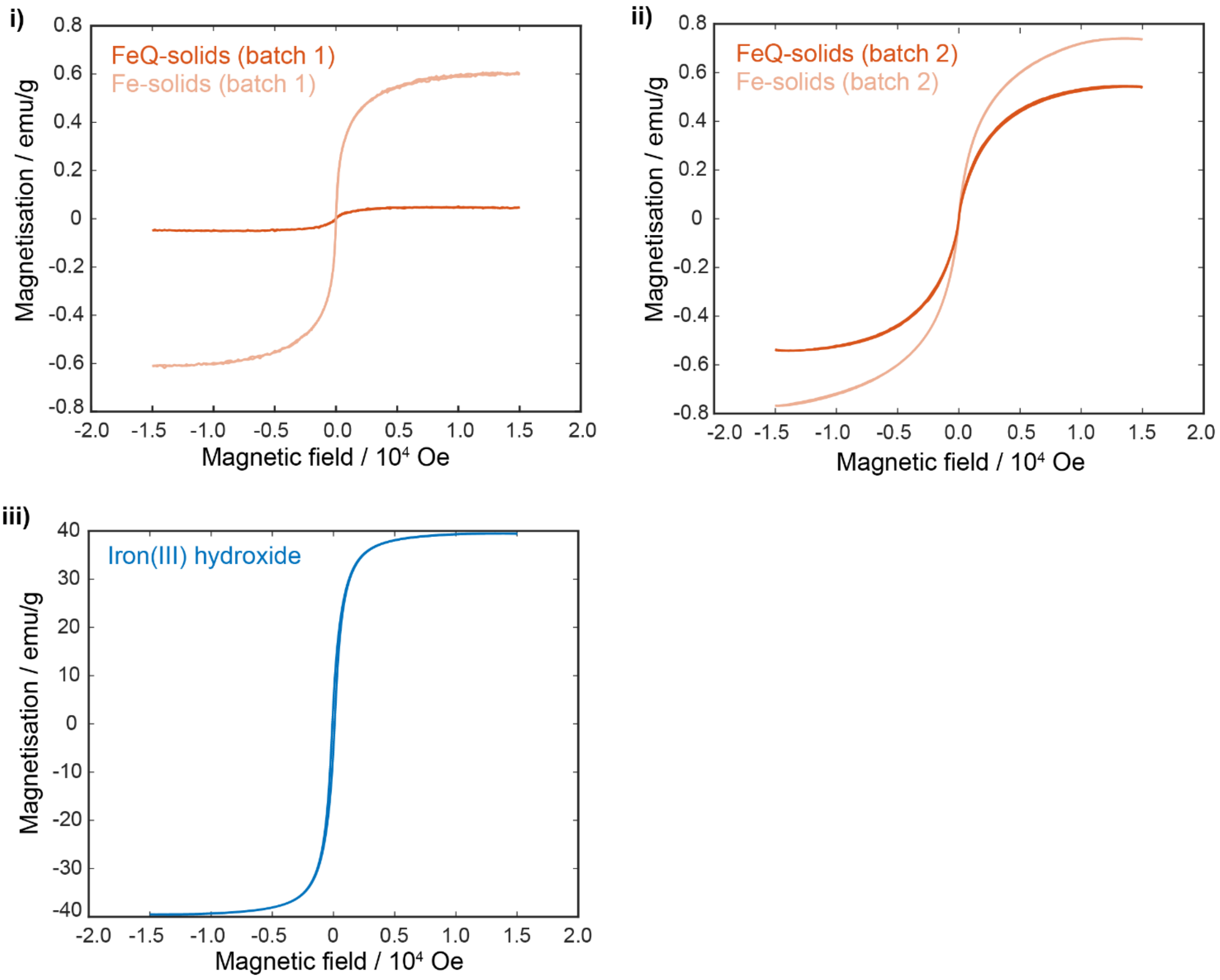

Figure S30: Hysteresis curves for the orange-brown precipitates measured by VSM. The hysteresis curves are shown for i) FeQ-solids and Fe-solids (batch 1), ii) FeQ-solids and Fe-solids (batch 2) and iii) iron(III) hydroxide standard. Note that only ~1mg of sample was available for the measurements on batch 1, which could impact data quality.

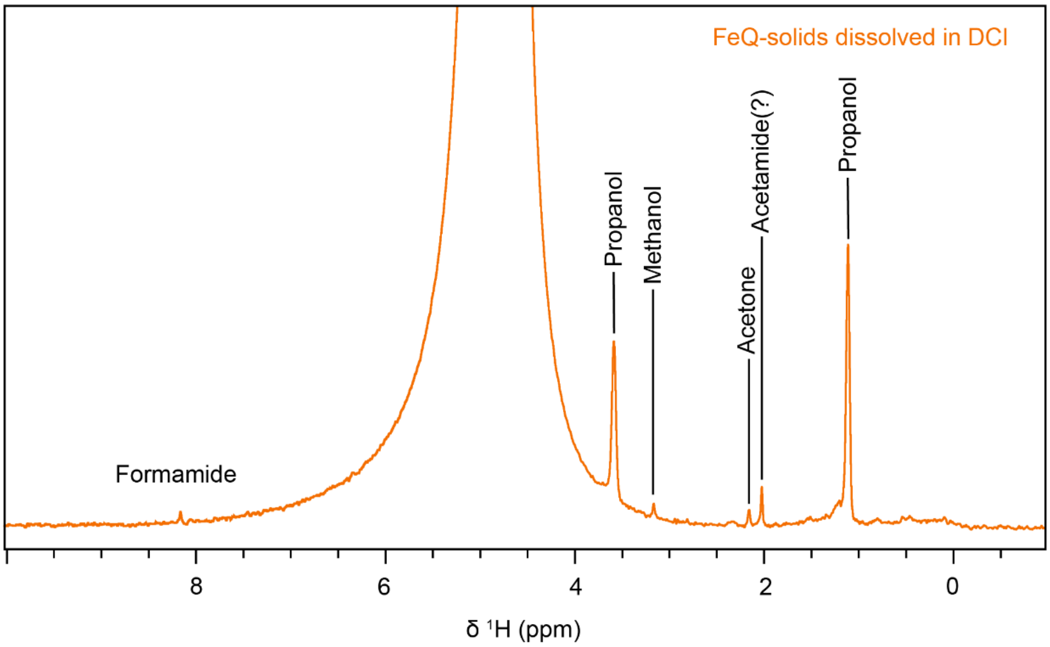

Figure S31: ^{1}H NMR of the FeQ-solids dissolved in DCl, collected at 9.4 T.

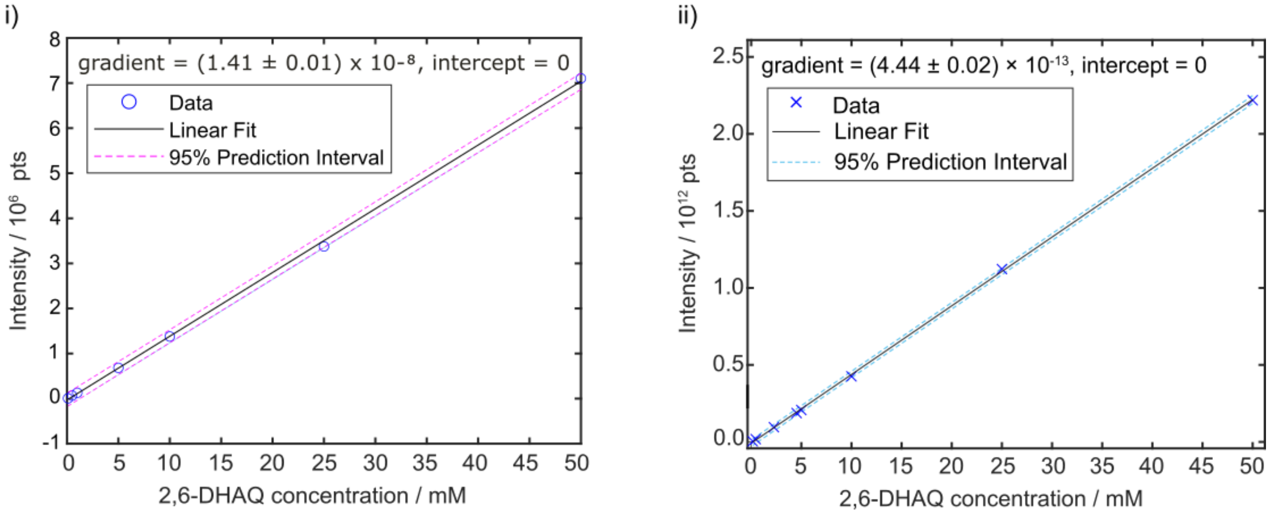

Figure S32: Calibration curves for 2,6-DHAQ H_A. The signal intensity for 2,6-DHAQ H_A was calibrated against 2,6-DHAQ concentrations for two different spectrometers. i) shows the calibration data for the 400 MHz standard bore Bruker magnet equipped with a 5 mm HD Smart Probe and Bruker Avance III spectrometer (used for *ex situ* simulated-crossover experiments and the full-cell crossover study). ii) shows the calibration data for the 400 MHz (9.4 T) wide-bore Bruker NMR spectrometer, equipped with a BBO 400 MHz W1S2 5mm Z-gradient diffusion probe (used for on-line simulated-crossover experiments). Panel ii) is adapted from a figure by Latchem *et al.*[1]

Table S1: Mean ^1H NMR chemical shift for the water peak (HOD) measured during *ex situ* simulated-crossover experiments. The mean and standard error were calculated from three repeat measurements.

	Sample	Mean HOD chemcial shift / ppm	Standard deviation	Standard error	Percentage error
Fresh	Ferricyanide	7.270	0.026	0.015	0.21%
	2,6-DPPEAQ + ferricyanide	7.213	0.017	0.012	0.16%
	2,6-DHAQ + ferricyanide	7.178	0.017	0.010	0.13%
	2,7-AQDS + ferricyanide	7.216	0.015	0.009	0.12%
	DHPS + ferricyanide	7.062	0.024	0.014	0.20%
1 week aged	Ferricyanide	7.252	0.015	0.009	0.12%
	2,6-DPPEAQ + ferricyanide	7.192	0.019	0.014	0.19%
	2,6-DHAQ + ferricyanide	6.733	0.024	0.014	0.21%
	2,7-AQDS + ferricyanide	7.175	0.018	0.011	0.15%
	DHPS + ferricyanide	6.973	0.016	0.009	0.13%

Table S2: Mean percentage loss of ferricyanide measured indirectly from changes in HOD ^1H NMR chemical shift during *ex situ* simulated-crossover experiments. The mean and standard error were calculated from three repeat measurements. The initial loss is the difference in ferricyanide concentration in the fresh catholyte relative to the spiked catholytes. The decrease in ferricyanide concentration due to volume changes has been accounted for.

Sample	Change in ferricyanide concentration / %			
	Initial change (after addition of anolyte)	Percentage error	Total change (1 week aging)	Percentage error
Ferricyanide	n/a	n/a	0.72%	0.24%
2,6-DPPEAQ + ferricyanide	0.70%	0.16%	1.58%	0.25%
2,6-DHAQ + ferricyanide	2.15%	0.13%	20.13%	0.25%
2,7-AQDS + ferricyanide	0.60%	0.12%	2.24%	0.19%
DHPS + ferricyanide	6.81%	0.20%	10.45%	0.24%

Table S3: Mean ^{1}H NMR integral for each anthraquinone anolyte measured in ferricyanide electrolyte during *ex situ* simulated-crossover experiments. The integral is taken from H_A, except for 2,7-AQDS where $(H_C + H_B)/2$ is used as H_A overlaps with formamide. The mean and standard error were calculated from three repeat measurements.

Sample		Mean H_A integral / a.u.	Standard deviation	Standard error	Percentage error
Fresh	2,6-DPPEAQ + ferricyanide	0.98	0.023	0.017	0.017
	2,6-DHAQ + ferricyanide	0.99	0.024	0.014	0.014
	2,7-AQDS + ferricyanide	0.72	0.020	0.011	0.016
1 week aged	2,6-DPPEAQ + ferricyanide	1.04	0.041	0.029	0.028
	2,6-DHAQ + ferricyanide	0.16	0.033	0.019	0.12
	2,7-AQDS + ferricyanide	0.59	0.035	0.020	0.034

Table S4: Mean percentage change in anthraquinone concentration measured during the *ex situ* simulated-crossover experiments. The percentage error was calculated from three repeat measurements.

Sample	Change in quinone concentration / %	
	Total change (1 week aging)	Percentage error
2,6-DPPEAQ + ferricyanide	6.90%	3.26%
2,6-DHAQ + ferricyanide	-84.32%	12.25%
2,7-AQDS + ferricyanide	-17.35%	3.79%

Table S5: Summary table comparing each type of crossover experiment used in this study.

Crossover experiment	Applied voltage	Apparatus	Electrolyte spiking	Total experiment duration	Temporal resolution of NMR analysis
**On-line full-cell experiment** (Experimental Methods 2)	Yes, up to 1.5 V	Fully operational redox flow battery, with on-line NMR flow tube on the catholyte side (flowing sample)	No (anolyte appears in catholyte due to real crossover)	~2 days	5 min
***Ex situ* simulated crossover experiment** (Experimental Methods 3)	No	Electrolytes stored in a sealed centrifuge vial, then sampled for *ex situ* NMR (static sample)	Yes (~ 2mM with anolyte of interest)	~1 week	1 week (1 spectrum collected before and after aging)
**On-line simulated crossover experiment** (Experimental Methods 4)	No	Electrolytes stored in a sealed electrolyte reservoir, with on-line NMR flow tube attached (flowing sample)	Yes (~ 2mM with anolyte of interest)	~2 days	5 min

Table S6: Combustion and ICP-MS elemental analysis of the orange-brown precipitates. The tables summarise the elemental analysis of the two batches of FeQ-solids and Fe-solids, and the iron(III) oxide and iron(III) hydroxide standards.

Sample	wt %			
	C	H	N	Fe
FeQ-solids (batch 1)	4.6	1.7	0.3	56
FeQ-solids (batch 2)	2.2	1.5	0.0	48
Fe-solids (batch 1)	5.8	1.8	0.1	48
Fe-solids (batch 2)	2.3	1.4	0.0	51
Iron(III) oxide standard (α-phase)	2.0	0.0	0.3	68
Iron(III) hydroxide standard (α-phase)	2.1	0.4	0.0	34

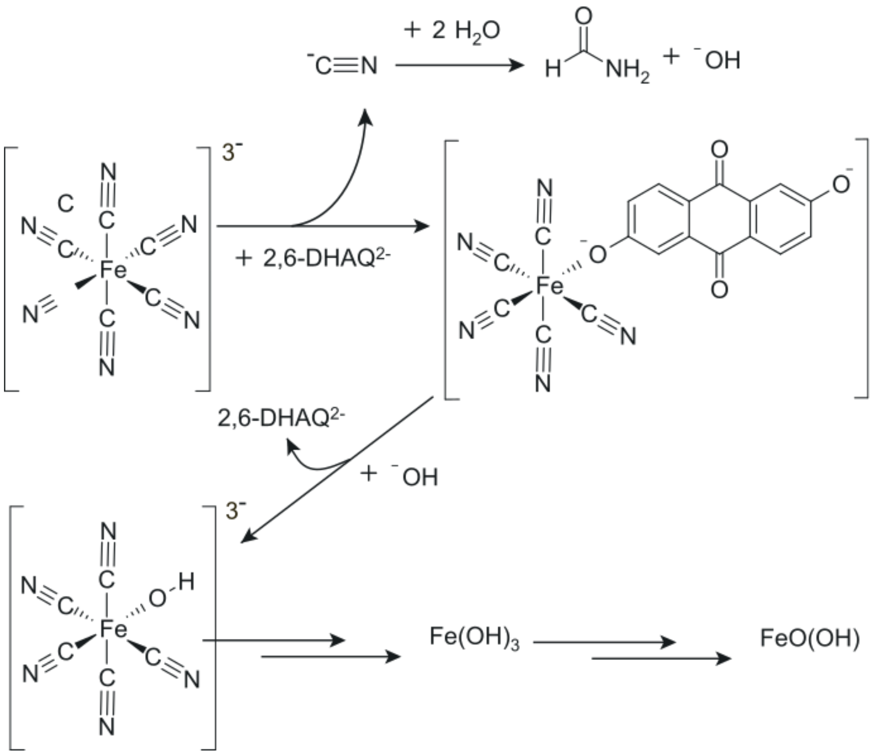

Scheme S1: Proposed mechanism of ferricyanide loss and conversion to iron oxide hydroxide precipitates in the presence of 2,6-DHAQ^{2-}.

References

(1) Latchem, E. J.; Kress, T.; Klusener, P. A. A.; Kumar, R. V.; Forse, A. C. Charge-Dependent Crossover in Aqueous Organic Redox Flow Batteries Revealed Using Online NMR Spectroscopy. *J. Phys. Chem. Lett.* **2024**, *15* (5), 1515–1520. https://doi.org/10.1021/acs.jpclett.3c03482.

(2) Hall, A. M. R.; Chouler, J. C.; Codina, A.; Gierth, P. T.; Lowe, J. P.; Hintermair, U. Practical Aspects of Real-Time Reaction Monitoring Using Multi-Nuclear High Resolution FlowNMR Spectroscopy. *Catal. Sci. Technol.* **2016**, *6* (24), 8406–8417. https://doi.org/10.1039/C6CY01754A.

(3) Zhao, E. W.; Liu, T.; Jónsson, E.; Lee, J.; Temprano, I.; Jethwa, R. B.; Wang, A.; Smith, H.; Carretero-González, J.; Song, Q.; Grey, C. P. In Situ NMR Metrology Reveals Reaction Mechanisms in Redox Flow Batteries. *Nature* **2020**, *579* (7798), 224–228. https://doi.org/10.1038/s41586-020-2081-7.

(4) Zhao, E. W.; Shellard, E. J. K.; Klusener, P. A. A.; Grey, C. P. *In Situ* Bulk Magnetization Measurements Reveal the State of Charge of Redox Flow Batteries. *Chem. Commun.* **2022**, *58* (9), 1342–1345. https://doi.org/10.1039/D1CC01895G.

(5) Evans, D. F. 400. The Determination of the Paramagnetic Susceptibility of Substances in Solution by Nuclear Magnetic Resonance. *J. Chem. Soc.* **1959**, 2003. https://doi.org/10.1039/jr9590002003.

(6) Altass, H. M.; Khder, A. E. R. S. Catalytic Oxidation of Carbon Monoxide over of Gold-Supported Iron Oxide Catalyst. *Materials Research Innovations* **2018**, *22* (2), 107–114. https://doi.org/10.1080/14328917.2016.1264707.
